# CDKL3 is a promising biomarker for diagnosis and prognosis prediction in patients with hepatocellular carcinoma

**DOI:** 10.3389/ebm.2024.10106

**Published:** 2024-06-27

**Authors:** Qingsi Wu, Mengran Lu, Huijuan Ouyang, Tingting Zhou, Jingyuan Lei, Panpan Wang, Wei Wang

**Affiliations:** ^1^ Department of Blood Transfusion, Second Affiliated Hospital of Anhui Medical University, Hefei, Anhui, China; ^2^ Anhui Provincial Key Laboratory of Microbiology and Parasitology, Hefei, Anhui, China; ^3^ School of Public Health, Department of Hygiene Inspection and Quarantine, Anhui Medical University, Hefei, Anhui, China; ^4^ Department of Gastroenterology, Yijishan Hospital of Wannan Medical College, Wuhu, Anhui, China

**Keywords:** CDKL3, biomarker, hepatocellular carcinoma, the cancer genome atlas, prognosis

## Abstract

Cyclin-dependent kinase-like 3 (CDKL3) has been identified as an oncogene in certain types of tumors. Nonetheless, its function in hepatocellular carcinoma (HCC) is poorly understood. In this study, we conducted a comprehensive analysis of CDKL3 based on data from the HCC cohort of The Cancer Genome Atlas (TCGA). Our analysis included gene expression, diagnosis, prognosis, functional enrichment, tumor microenvironment and metabolic characteristics, tumor burden, mRNA expression-based stemness, alternative splicing, and prediction of therapy response. Additionally, we performed a cell counting kit-8 assay, TdT-mediated dUTP nick-end Labeling staining, migration assay, wound healing assay, colony formation assay, and nude mouse experiments to confirm the functional relevance of CDKL3 in HCC. Our findings showed that CDKL3 was significantly upregulated in HCC patients compared to controls. Various bioinformatic analyses suggested that CDKL3 could serve as a potential marker for HCC diagnosis and prognosis. Furthermore, CDKL3 was found to be involved in various mechanisms linked to the development of HCC, including copy number variation, tumor burden, genomic heterogeneity, cancer stemness, and alternative splicing of CDKL3. Notably, CDKL3 was also closely correlated with tumor immune cell infiltration and the expression of immune checkpoint markers. Additionally, CDKL3 was shown to independently function as a risk predictor for overall survival in HCC patients by multivariate Cox regression analysis. Furthermore, the knockdown of CDKL3 significantly inhibited cell proliferation *in vitro* and *in vivo*, indicating its role as an oncogene in HCC. Taken together, our findings suggest that CDKL3 shows promise as a biomarker for the detection and treatment outcome prediction of HCC patients.

## Impact statement

Cyclin-dependent kinase-like 3 (CDKL3) has been reported as an oncogene in certain types of tumors. Nevertheless, its significance in hepatocellular carcinoma (HCC) has not been well investigated. This study demonstrated that CDKL3 was significantly upregulated in HCC patients compared to controls. Our analysis also showed that CDKL3 had independent prognostic value in HCC. Functional experiments further confirmed the oncogenic function of CDKL3 in HCC. Further research on the mechanisms underlying the function of CDKL3 in HCC is warranted.

## Introduction

Hepatocellular carcinoma (HCC) represents the predominant subtype among primary liver cancers, constituting a majority, exceeding 90% of total cases [1]. This malignancy is frequently linked to well-recognized risk factors, namely, metabolic syndrome, hepatitis B and C viral infections, diabetes mellitus, and alcohol abuse [1]. The complex nature of HCC, with its diverse presentations and tumor heterogeneity, makes it challenging to treat. Additionally, HCC has a worse prognosis compared to many other types of cancer, and its mortality and incidence rates continue to rise globally [2]. Unfortunately, the diagnoses of a significant number of HCC patients are made when the malignancy has already progressed to an advanced stage, leading to dismal survival outcomes due to increased recurrence rates [3]. Therefore, it is crucial to investigate potential therapeutic targets and diagnostic/prognostic biomarkers for HCC to improve patient outcomes.

Cyclin-dependent kinase-like 3 (CDKL3), also referred to as NKIAMRE, is a constituent of the cyclin-dependent protein kinase-like family [4, 5]. It is located on human chromosome 5q31.1 and encodes a 52 kDa protein consisting of 455 amino acids [6]. CDKL3 was first identified in 2001 and plays a role in cell proliferation [7]. Although it is expressed at low levels in all tissues, it appears to have an essential function in regulating the cell cycle of tumor cells [8, 9]. Recent studies have found abnormal expression of CDKL3 in several cancers, particularly osteosarcoma, esophageal squamous cell carcinoma, colorectal cancer, glioma, and cholangiocarcinoma [6, 8, 10–12]. This abnormal expression is closely associated with tumor occurrence, development, and prognosis [13].

While some new diagnostic and prognostic markers for HCC have been reported in recent years [14, 15], research on CDKL3 in the context of tumors remains limited. Previous studies have primarily focused on diseases of the central nervous system [16–18]. However, a recent study by Sun et al. indicated that exosomal microRNA (miRNA)-205-5p from bone marrow mesenchymal stem cells can inhibit liver cancer, partially through the knockdown of CDKL3 [19]. Yet the role of CDKL3 in HCC has not been explored comprehensively. In this study, we utilized publicly available RNA sequencing (RNA-seq) data to examine the impact of CDKL3 in HCC patients. Furthermore, we conducted *in vitro* and *in vivo* functional assays to elucidate the potential biological function of CDKL3 in HCC.

## Materials and methods

### Data processing

The RNA-seq data consisting of copy number variation (CNV) data, messenger RNAs (mRNAs), long non-coding RNAs (lncRNAs), somatic mutation data (MuTect2), miRNAs, as well as clinicopathological information of HCC patients were obtained from The Cancer Genome Atlas (TCGA) database (LIHC dataset). The data were collected in December 2021 and preprocessed using the “TCGAbiolinks” package [20]. Additionally, DNA methylation data were acquired from UCSC Xena^1^ and underwent preprocessing procedures consistent with previously documented methodologies [21]. The median beta value corresponding to the CDKL3 gene probes was mapped to its promoter, including 1stExon, 5′ untranslated region, transcription start site (TSS)200, and TSS1500 [22]. The mRNA and lncRNA data, originally presented in fragments per kilobase per million reads (FPKM), underwent a conversion to transcripts per kilobase million (TPM) values, followed by a subsequent log_2_ conversion. The mature miRNA (mirbase version 21) data, initially in row count values, were transmuted into reads per million mapped reads (RPM) and subjected to log2 conversion. Inclusion criteria for this study were patients diagnosed with primary solid HCC who were 18 years or older. Exclusion criteria included a history of preoperative adjuvant therapy, lack of survival time data, the recorded survival time <30 days, or multiple samples from a single patient [23]. A total of 330 HCC patients and 47 controls were ultimately enrolled. The clinicopathological information of the HCC patients can be found in Supplementary Table S1.

### Expression and prognosis analyses

The investigation into pan-cancer expression profiles was facilitated through the TIMER 2.0 tool^2^ [24]. The expression difference of CDKL3 between HCC patients and controls in the LIHC dataset was analyzed. Kaplan-Meier (KM) overall survival (OS) analysis with the log-rank test was conducted by dividing HCC patients into the high-CDKL3 (hCDKL3) group and the low-CDKL3 (lCDKL3) group. This division was based on the optimal cut-off expression values determined using the maximally selected rank statistics algorithm. The univariate Cox regression model was applied for survival analysis, and the expression difference of CDKL3 between the hCDKL3 and lCDKL3 groups was investigated. Multivariate Cox analysis was implemented to assess the independence of CDKL3 for OS prediction. Furthermore, a nomogram incorporating other independent parameters for OS prediction was established. Internal validation was executed by computing the adjusted Harrell’s concordance index (C-index) utilizing the bootstrapping approach with 1,000 resamples [23]. The performance of the independent parameters of OS and the nomogram were evaluated using the areas under the curve (AUC) values, decision curve analysis (DCA), and calibration curves [25–27].

### Bioinformatic analysis

To investigate the potential difference in biological function between the two groups, a gene set enrichment analysis (GSEA) was undertaken utilizing the Kyoto Encyclopedia of Genes and Genomes (KEGG) and HALLMARK gene sets derived from the Molecular Signatures Database (MSigDB^3^) [28]. To establish significance, we conducted a screening process that involved identifying *p* values < 0.05 and a false discovery rate >0.25. Furthermore, the functional annotation of genes in the KEGG pathway was performed using the online tool KOBAS-i^4^ [29]. Single-sample GSEA (ssGSEA) was performed to explore the tumor microenvironment (TME) and metabolic characteristics using 29 functional gene expression signatures (Fges), which were recently proposed by Bagaev et al. [30]. Moreover, gene set variation analysis was employed to analyze metabolism-associated signatures, as detailed in established methods [31]. Immune checkpoint gene (ICG) expression discrepancies between the two groups were also analyzed. Somatic copy number alternation (SCNA) burden was assessed, representing the gain or depletion of total gene count at the focal and arm levels. This analysis was carried out using the GISTIC 2.0 module^5^ [32, 33]. The calculation of tumor mutation burden (TMB) for every patient adhered to the methodology outlined in prior descriptions [34]. The mRNA expression-based stemness index (mRNAsi) for HCC patients was obtained from a published reference [35]. Additionally, the percent splice-in (PSI) value of CDKL3 was retrieved from the SpliceSeq database using default parameters^6^ [36]. For the missing PSI values, the average value of each event was used to fill them up. Only events with a mean PSI value greater than 0.1 were retained for further analysis in this study. The details for analyzing competitive endogenous RNA regulatory networks and estimating the benefits of immunotherapy and chemotherapy can be found in the Supplementary Material.

### Cell culture, transfection, quantitative real-time polymerase chain reaction (qPCR), and Western blot assays

The human HCC cell lines SMMC-7721 and HepG2 were acquired from GeneChem Corporation (Shanghai, China) and the Cell Bank of Type Culture Collection of the Chinese Academy of Sciences (Shanghai, China), respectively. Subsequently, RPMI-1640 (Gibco, Waltham, MA, United States) or Dulbecco’s modified Eagle’s medium (DMEM) added with 10% heat-inactivated fetal calf serum (FBS, Gibco, Thermo Fisher Scientific, Inc.) was used to culture these cell lines at 37°C. Concentrated and purified lentiviral particles expressing a short hairpin RNA (shRNA) were purchased from GeneChem Corporation (Shanghai, China). To perform transfection, the control lentivirus (shCtrl) or the target lentivirus (shCDKL3) was added to tumor cells (7 × 10^4^ cells/well) in a six-well plate (Corning Incorporated, United States). After 72 h, the expression level of CDKL3 was assessed using qPCR and Western blot assays. More detailed information regarding the qPCR and Western blot assays can be found in the Supplementary Material.

### Cell proliferation, colony formation, and apoptosis assays

The HCC cells, following infection, were placed in a 96-well plate (Corning Incorporated, United States) and subsequently cultured for a span of 4 days. To assess cellular proliferative capacity, we performed a daily cell counting kit-8 (CCK-8) assay (Beyotime’s kit from Shanghai, China) as per package guidelines [37]. A colony formation experiment involved plating 2,000 infected cells per well over a 6-well plate. After 2 weeks of incubation, 4% paraformaldehyde solution (Beyotime, Shanghai, China) was employed to fix the tumor cells for 30 min. Subsequently, crystal violet solution (Beyotime, Shanghai, China) at 0.1% was utilized to stain the cells for 20 min. Under a light microscopy, the number of viable colonies (>50 cells/colony) was determined. To perform the apoptosis assay, we plated the infected cells on prepositioned slides in a 12-well plate (Corning Incorporated, United States). After an overnight incubation, the cells were immobilized with a 4% paraformaldehyde solution for 30 min. The nuclei were stained using DAPI (Beyotime, Shanghai, China). The detection of cell apoptotic rate was carried out utilizing a TdT-mediated dUTP nick-end labeling (TUNEL) kit (Beyotime, Shanghai, China), as described previously [38].

### Tumour xenograft assay

For the tumor xenograft assay, six BALB/C nude mice (Henan Scrobes Company, China), aged 5 weeks, were used in each group (*n* = 6/group). At the Center for Laboratory Animal Research in Anhui Medical University, the mice were housed in a pathogen-free environment. All experiments with animals were done in compliance with institutional guidelines and with appropriate oversight. The Experimental Animal Ethics Committee of Anhui Medical University approved this study (No. 20200695). Subcutaneous transplantation of SMMC-7721 cells stably expressing shControl or shCDKL3 (3×10^6^ cells) was performed on the upper right back of the mice. The size of the tumor (computed as: [(π/6) × (length) × (width) [2]]) and its weight were measured every 4 days over a duration of 4 weeks until the humane euthanasia of mice.

### Statistical analysis

Categorical data were compared utilizing the chi-square or Fisher’s exact test, whereas the Wilcoxon rank-sum test or Kruskal‒Wallis test was employed for the comparison of continuous variables. The correlation was tested using a rank correlation method, either Pearson’s or Spearman’s. R 4.0.1 (The R Foundation for Statistical Computing, Vienna, Austria) and GraphPad Prism 8.0.2 (GraphPad Software, Inc.) were used to perform all statistical tests. Statistical significance was assumed when the *p*-value was <0.05 (two-tailed) unless otherwise specified.

## Results

### CDKL3 is a potential prognostic factor for HCC patients

**FIGURE 1:**
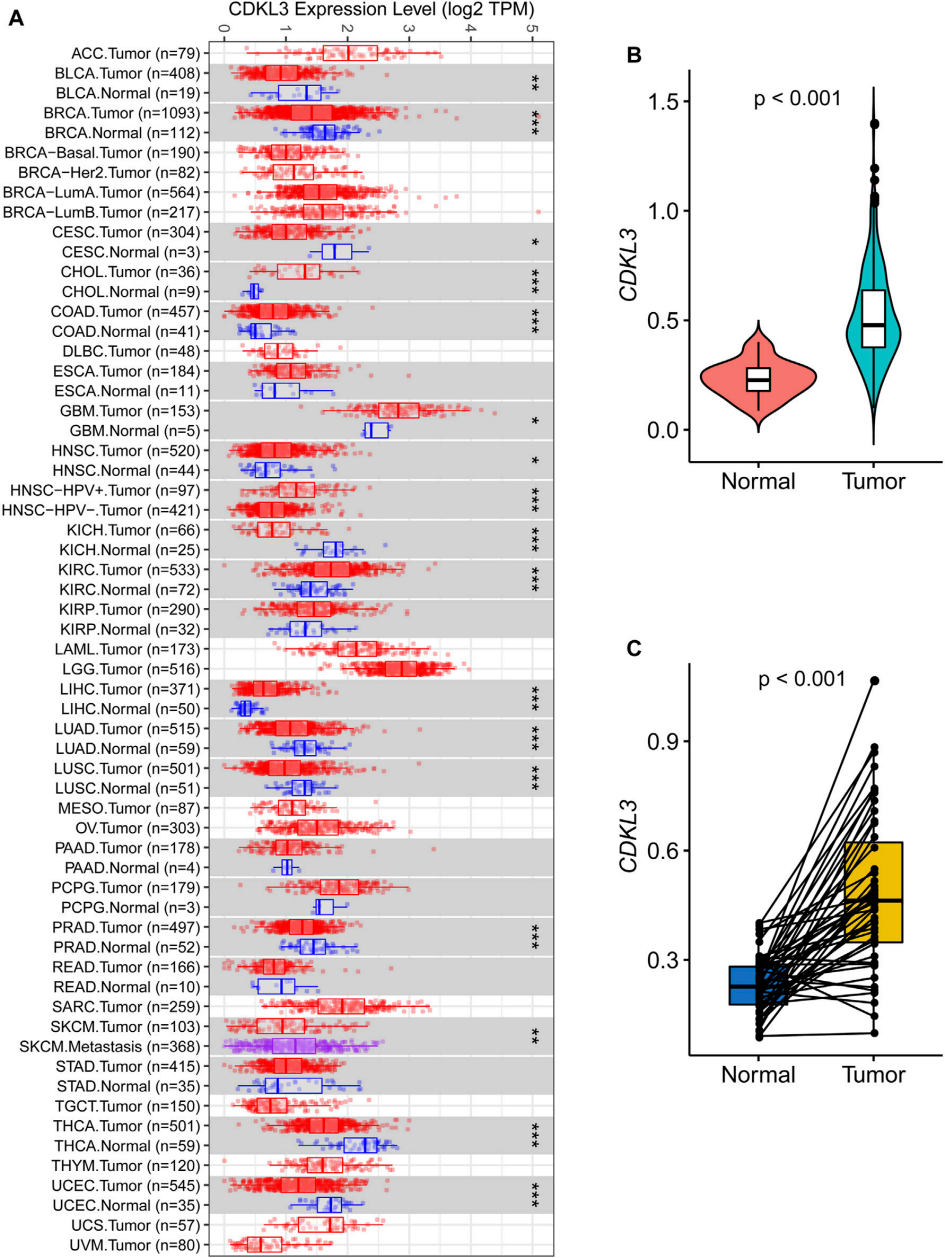
Expression of CDKL3 in HCC. **(A)** The expression of CDKL3 in Pan-cancer based on TCGA data via Tumor IMmune Estimation Resource 2.0 (TIMER 2.0, http://timer.comp-genomics.org/). Comparative CDKL3 expression levels in HCC and adjacent normal tissue: plots of unpaired **(B)** and paired **(C)** data. HCC, hepatocellular carcinoma.

**FIGURE 2:**
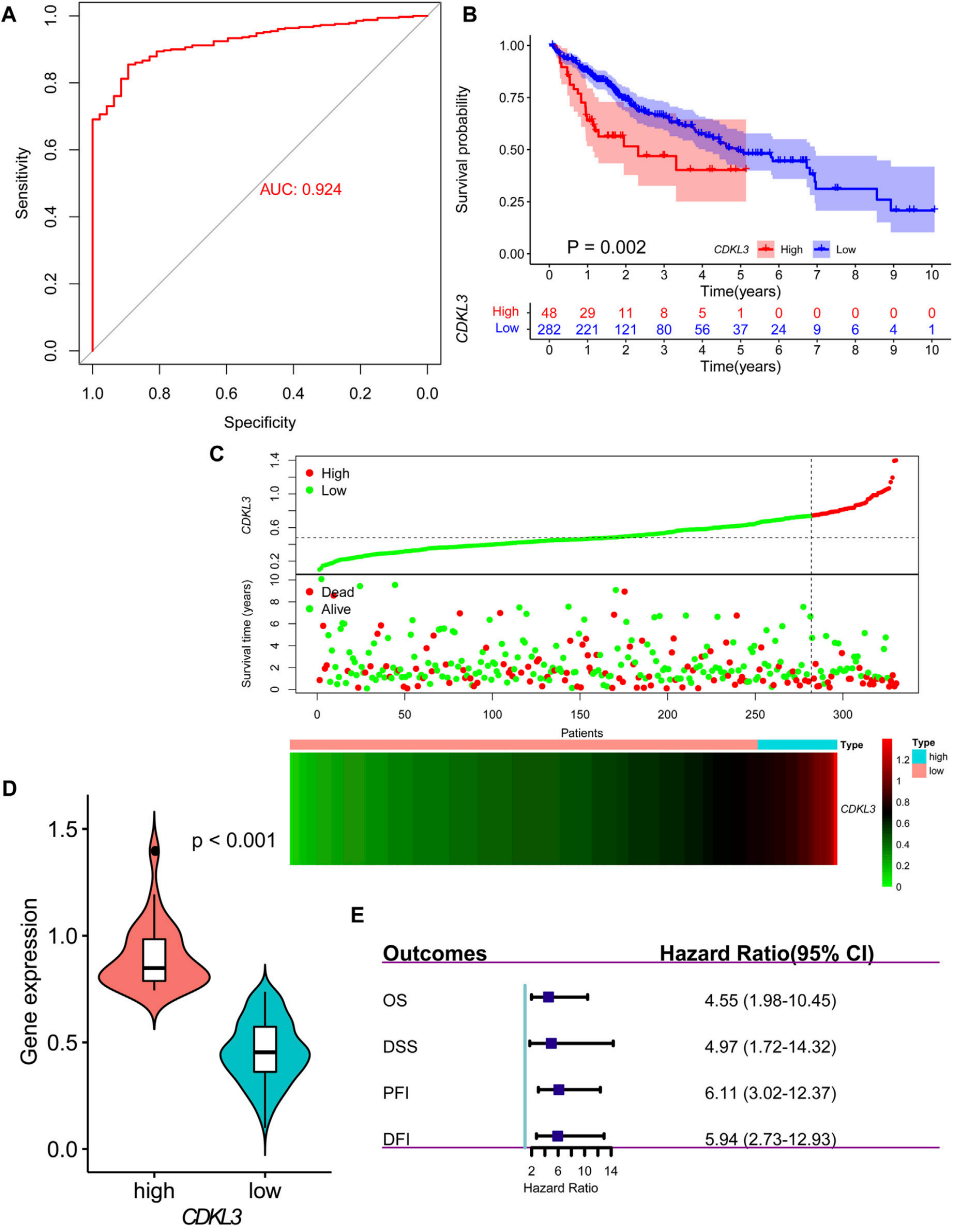
Diagnosis and prognosis of CDKL3 in HCC. **(A)** ROC curve for CDKL3 expression in HCC. **(B)** Kaplan-Meier overall survival curves of hCDKL3 and lCDKL3 and the survival difference was evaluated by log-rank test. **(C)** The dot plot and heatmap demonstrating the survival status and expression of CDKL3 in hCDKL3 and lCDKL3, respectively. **(D)** The expression of CDKL3 was compared between hCDKL3 and lCDKL3. **(E)** Forest plot showing the results of univariate Cox analyses for overall survival, disease-specific survival, progression-free interval and disease-free interval. HCC, hepatocellular carcinoma; hCDKL3, high-CDKL3 group; lCDKL3, low-CDKL3 group.

Analysis of data from TIMER 2.0 revealed that CDKL3 was significantly dysregulated in various solid tumors (Figure 1A). In the TCGA dataset, both unpaired and paired analyses demonstrated markedly upregulated levels of CDKL3 in HCC patients compared to controls (Figures 1B, C). Notably, CDKL3 displayed excellent diagnostic performance for HCC, with an impressive AUC value of 0.924 (Figure 2A). KM analysis revealed that the OS of the hCDKL3 group was lower than the lCDKL3 group (Figures 2B, C). Consistent results were observed for progression-free interval (PFI), disease-free interval (DFI), and disease-specific survival (DSS) (Supplementary Figure S1). Figure 2D illustrates the gene expression differences between the hCDKL3 and lCDKL3 groups. Univariate Cox analysis highlighted the association of CDKL3 level with OS, DSS, DFI, and PFI (Figure 2E). Additionally, we explored how CDKL3 expression related to clinicopathological variables (like tumor grade and family history of cancer), and found a substantial link between the two (Supplementary Figure S2).

### Construction of a survival nomogram

**FIGURE 3:**
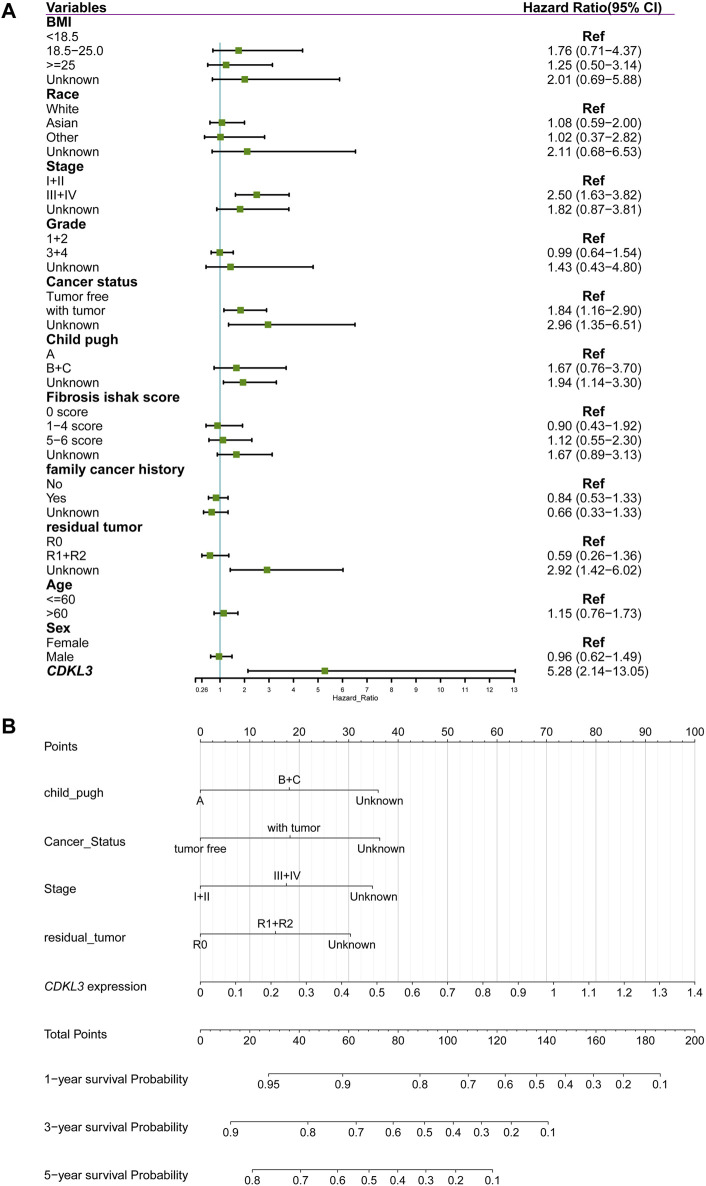
Construction of nomogram for predicting overall survival of hepatocellular carcinoma in TCGA cohort. **(A)** Forest plot showing the results of multivariate Cox analysis. **(B)** Nomogram.

CDKL3 was shown to independently function as a predictor of OS in a multivariate Cox regression study (Figure 3A). To aid in clinical decision-making, a nomogram was devised incorporating CDKL3 level and other independent clinicopathological variables (Figure 3B). The predicted and observed survival rates were highly consistent, as shown by calibration curve graphs (Supplementary Figure S3A). Receiver operating characteristic (ROC) curve analysis illustrated that the nomogram outperformed individual factors alone in estimating 1-, 3-, and 5-year OS, as indicated by higher AUC values (0.78, 0.75, and 0.78, respectively) (Supplementary Figure S3B). Furthermore, DCA demonstrated that the nomogram yielded greater net benefits compared to individual factors (Supplementary Figure S3C). Internal validation yielded an adjusted C-index of 0.713, indicating the reliability of the nomogram in prognostic prediction.

### Genetic features between the hCDKL3 and lCDKL3

**FIGURE 4:**
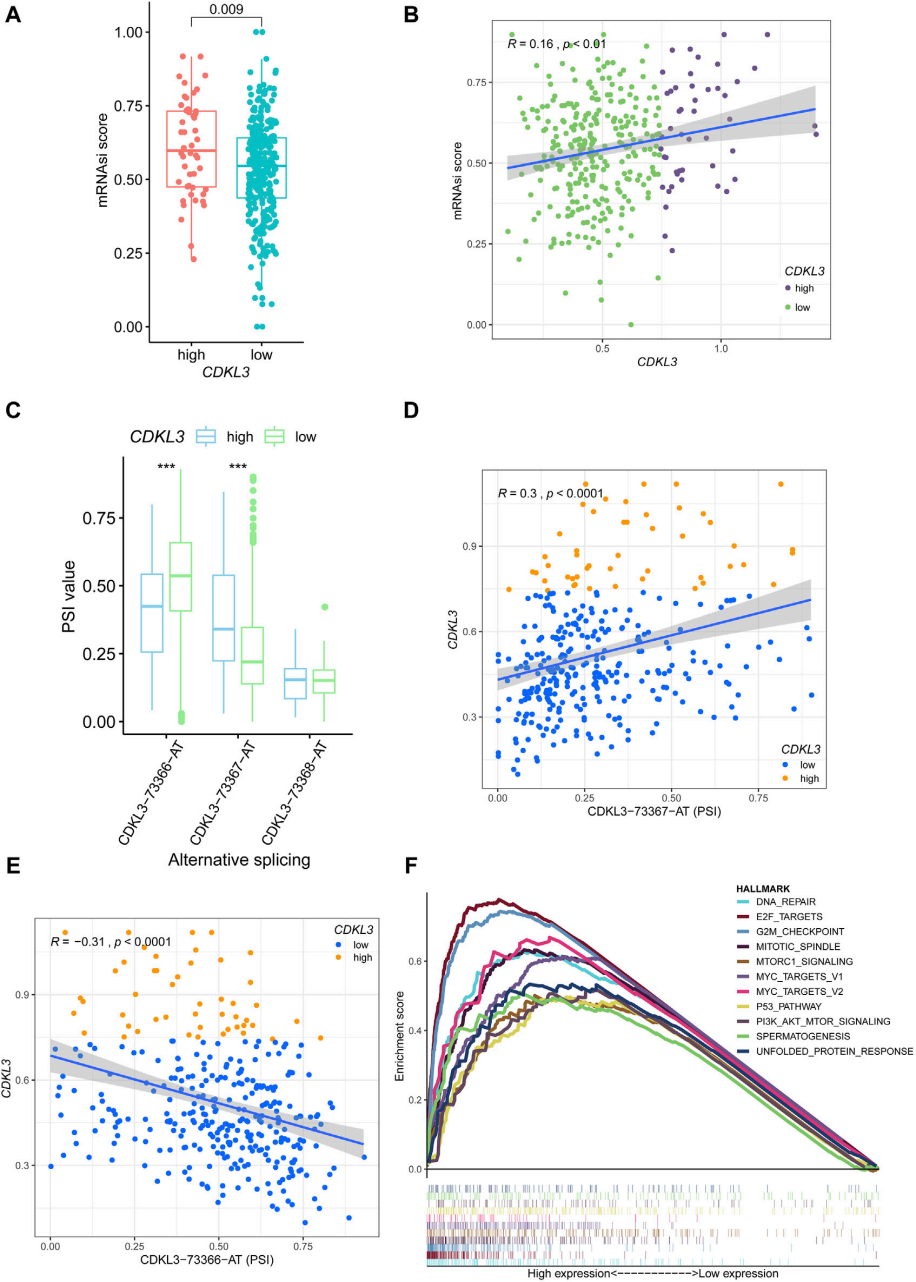
Genetic features of CDKL3 in HCC. **(A)** The mRNAsi score was compared between hCDKL3 and lCDKL3. **(B)** The relationship between the CDKL3 expression and mRNAsi score. **(C)** The PSI values of three alternative splicings were compared between hCDKL3 and lCDKL3. **(D)** The relationship between CDKL3 expression and PSI values of CDKL3-73367-AT. **(E)** The relationship between CDKL3 expression and PSI values of CDKL3-73366-AT. **(F)** The hallmarks of tumor sets were enriched in hCDKL3 using GSEA. ***p < 0.001. HCC, hepatocellular carcinoma; hCDKL3, high-CDKL3 group; lCDKL3, low-CDKL3 group; PSI, percent splice-in.

The Supplementary Figures S4, S5 presented the mutation-related findings of CDKL3. In terms of the mRNAsi, the hCDKL3 group exhibited a score superior to that of the lCDKL3 cohort (Figure 4A). Furthermore, the CDKL3 expression was positively linked to the mRNAsi score (Figure 4B). Figure 4C demonstrated three alternative splicing events for CDKL3. The hCDKL3 group displayed a higher PSI value for CDKL3−73367−AT and a lower PSI value for CDKL3−73366−AT when compared to the lCDKL3 group. Additionally, the expression of CDKL3 had a positive correlation with the PSI value of CDKL3−73367−AT and a negative correlation with the PSI value of CDKL3−73366−AT (Figures 4D, E). Interestingly, Figure 4F revealed that the hCDKL3 group was significantly enriched in hallmark pathways associated with tumorigenesis, especially the G2/M checkpoint, E2F targets, DNA repair, MYC targets V1 and V2, the P53 pathway, and PI3K−AKT−MTOR signaling. These findings strongly suggest that the hCDKL3 and lCDKL3 groups exhibit distinct genetic features.

### TME and metabolism characteristics of the hCDKL3 and lCDKL3 groups

**FIGURE 5:**
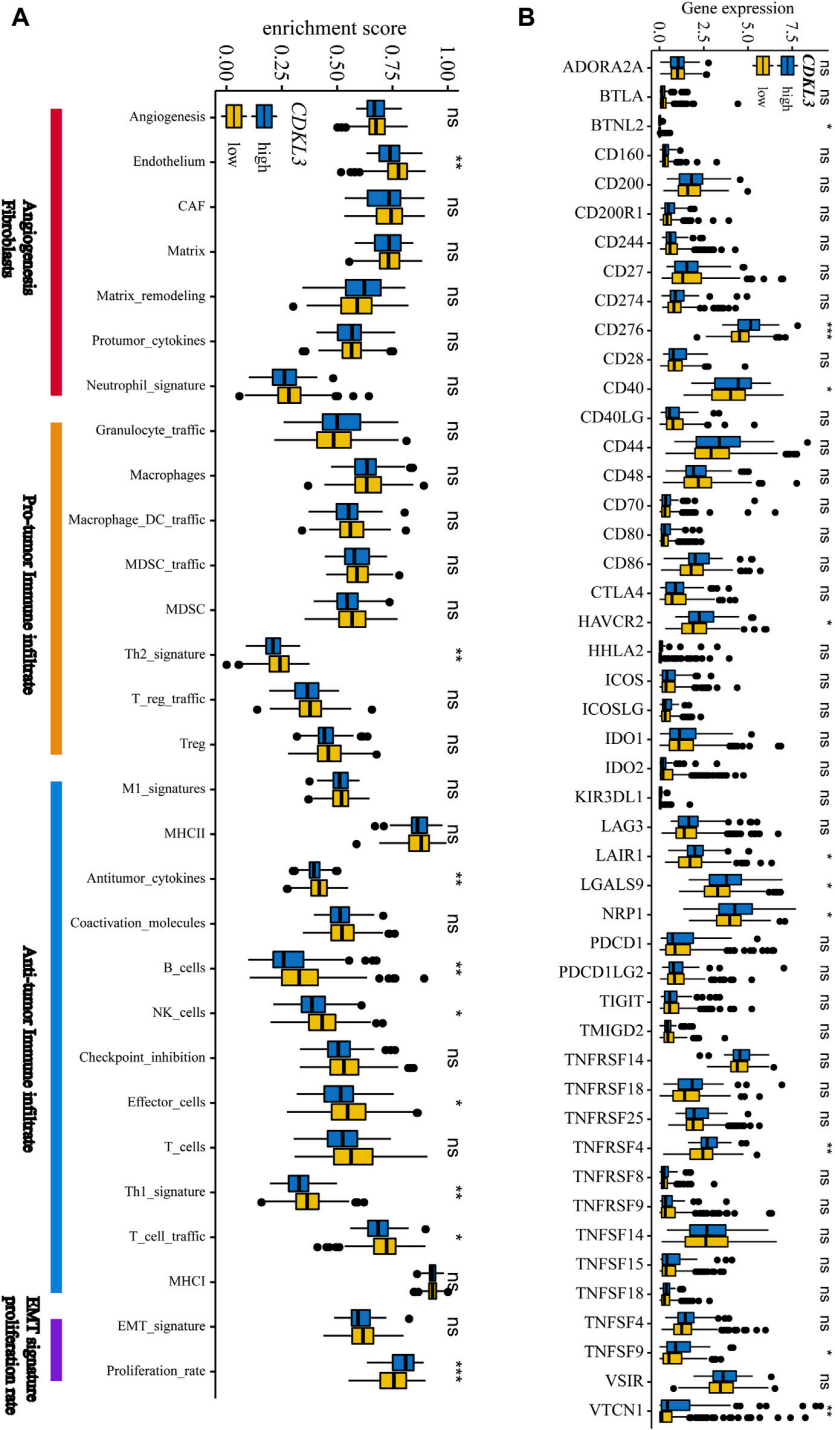
The tumour microenvironment characteristics of hCDKL3 and lCDKL3. **(A)** ssGSEA enrichment score of 29 Fges in hCDKL3 and lCDKL3. **(B)** Expression levels of immunosuppression-related molecules in hCDKL3 and lCDKL3. *p < 0.05, **p < 0.01, ***p < 0.001, ns, not significantly significant. hCDKL3, high-CDKL3 group; lCDKL3, low-CDKL3 group; ssGSEA, single-sample gene set enrichment analysis.

**FIGURE 6:**
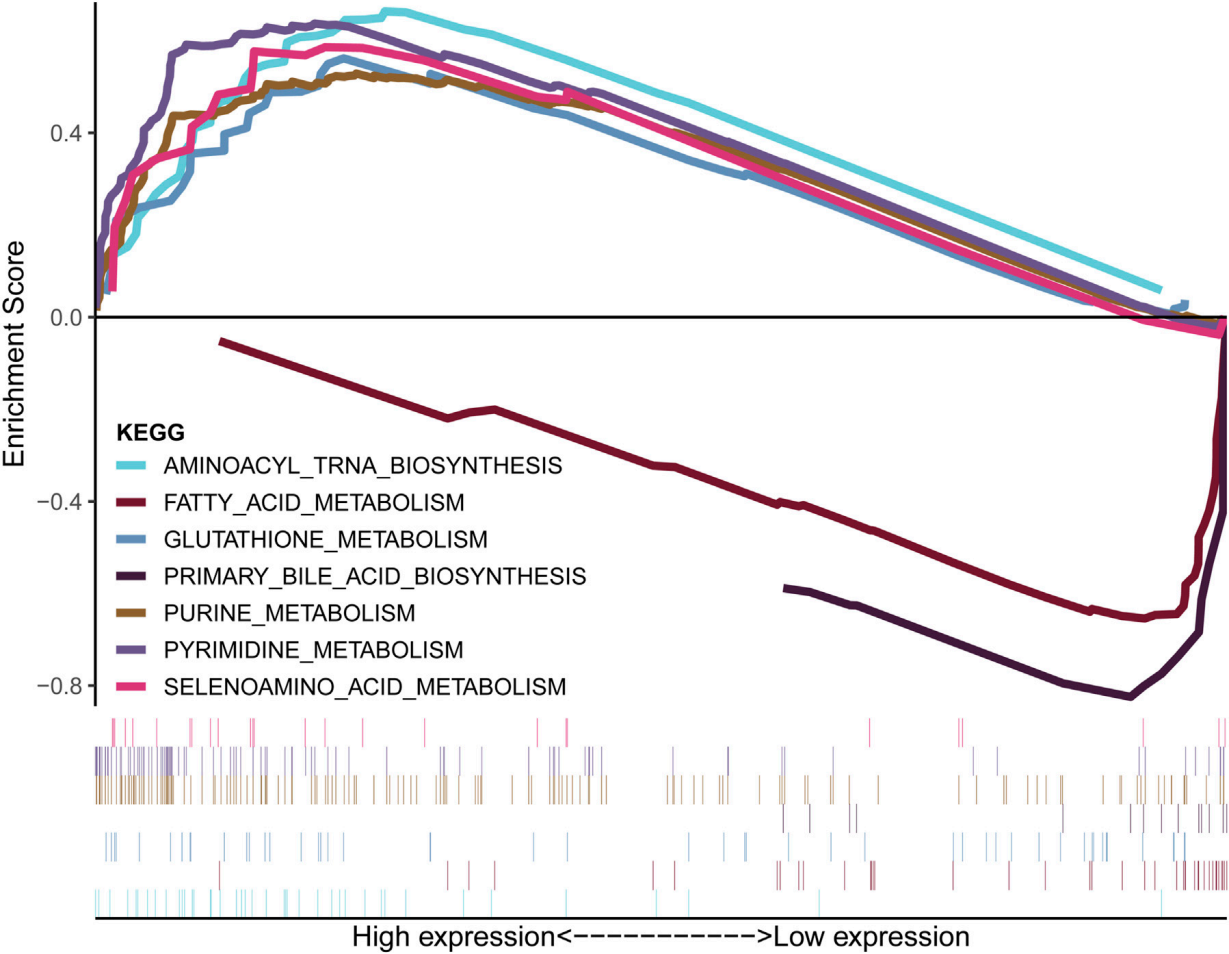
Metabolism-related KEGG pathways were enriched, as demonstrated using GSEA. KEGG: Kyoto Encyclopedia of Genes and Genomes; GSEA: gene set enrichment analysis.

Figure 5A demonstrates that the lCDKL3 group is characterized by a significantly decreased enrichment score of the proliferation rate signature and a notably elevated enrichment score of Fges, which is related to the cluster of antitumor immune infiltrates. This enrichment included antitumor cytokines, B cells, NK cells, effector cells, the Th1 signature, and T-cell traffic signatures. Correlation analysis confirmed a positive link between CDKL3 expression and the enrichment score of the proliferation rate signature, and a negative correlation with the enrichment score of antitumor cytokines, B cells, NK cells, effector cells, the Th1 signature, and T-cell traffic signatures (Supplementary Figure S6). Moreover, an investigation into the difference in T-cell exhaustion markers between the lCDKL3 and hCDKL3 groups highlighted that hCDKL3 was associated with increased expression of markers such as BTNL2, CD276, CD40, HAVCR2, LAIR1, LGALS9, NRP1, TNFRSF4, TNFSF9, and VTCN1 (Figure 5B). Additionally, the results of GSEA of KEGG pathways indicated that both the lCDKL3 and hCDKL3 groups were significantly enriched in multiple metabolic pathways (Figure 6). Further exploration of the enrichment scores of metabolism-associated pathways in the four categories (amino acids, carbohydrates, lipids, and others) between the lCDKL3 and hCDKL3 groups revealed significant differences. Notably, the hCDKL3 group exhibited significantly decreased pathway enrichment scores for lipid and other pathways. Similar patterns were observed in most amino acid and carbohydrate pathways, except for the GLUCOSE, PURINE, PYRIMIDINE, and SELENOAMINO ACID metabolism pathways (Supplementary Figures S7A–D). Additionally, the CDKL3 levels were inversely linked to the enrichment score of most differentially enriched pathways (Supplementary Figure S7E). Altogether, these findings suggest that the lCDKL3 and hCDKL3 groups possess distinct TME and metabolism characteristics.

### Functional annotations and tumour immune single-cell analysis

We conducted a thorough screening of 50 protein-coding genes that showed a strong positive correlation with CDKL3, as well as 50 genes that displayed a negative correlation (Supplementary Table S2). Utilizing KOBAS-i, we executed functional annotation of these genes in the KEGG pathway, and observed significant enrichment in various clusters and functions (Supplementary Figure S8). Specifically, these genes were found to be closely associated with metabolism pathways, the TGF-beta signalling pathway, and the PPAR signalling pathway, all of which were considered to impart a substantial role in the growth of HCC (adjusted *p*-value <0.05). For further analysis, we turned to the TISCH database, where we conducted a single-cell analysis to assess the expression of CDKL3 in different cell types. However, our findings did not reveal any significant differences in CDKL3 expression among these cell types (Supplementary Figure S9). Additional details regarding these results can be found in the Supplementary Material. The results for analyzing competitive endogenous RNA regulatory networks and estimating the benefits of immunotherapy and chemotherapy can be found in the Supplementary Figures S10, S11.

### Verification of the function of CDKL3 *in vitro* and *in vivo*

**FIGURE 7:**
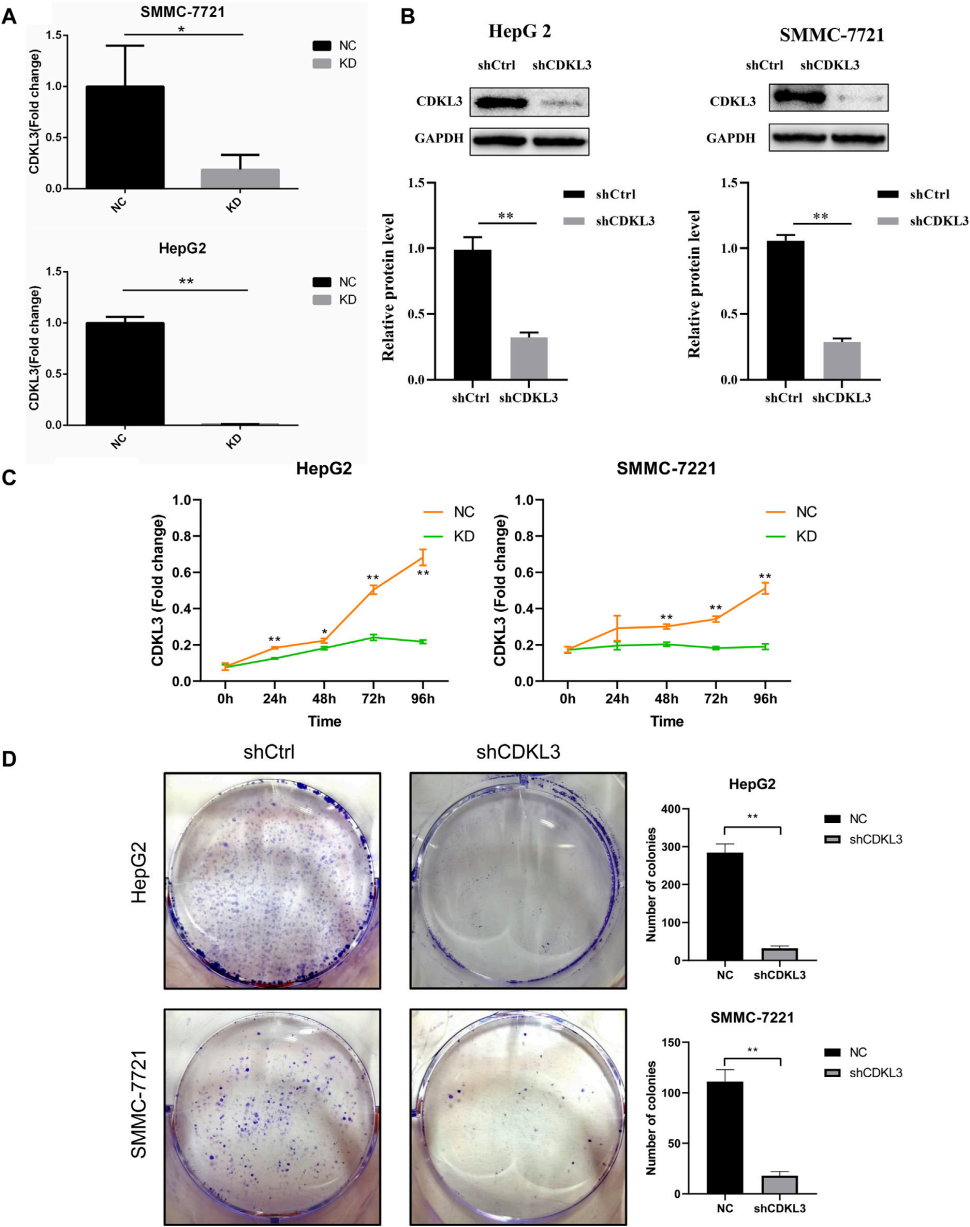
Verification of CDKL3 function *in vitro*. **(A)** qRT-PCR and **(B)** western blot assays were performed to evaluate the expression level of CDKL3 after transfection by shCDKL3 in hepatocellular carcinoma cell lines. **(C)** Cell viability was determined using CCK8 assays. **(D)** Colony formations assays were performed to evaluate cell proliferation ability. *p < 0.05, **p < 0.01. Error bars indicate mean ± SD. qRT-PCR, quantitative real-time polymerase chain reaction; CCK8, Cell Counting Kit-8.

**FIGURE 8:**
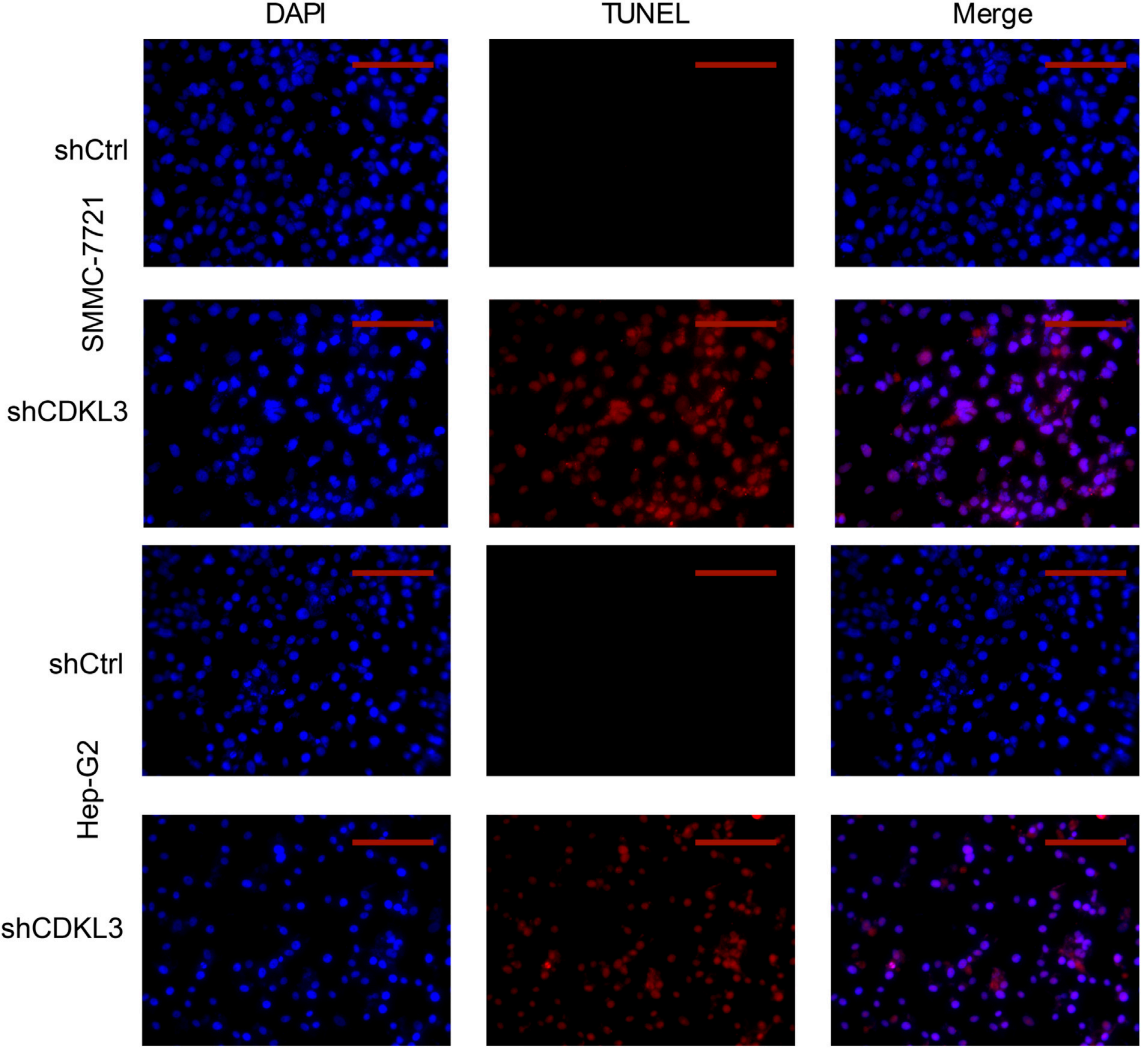
TUNEL (Scale bar, 400 μm) assay was performed to detect cell apoptosis. TUNEL, TdT-mediated dUTP Nick-End Labeling.

**FIGURE 9:**
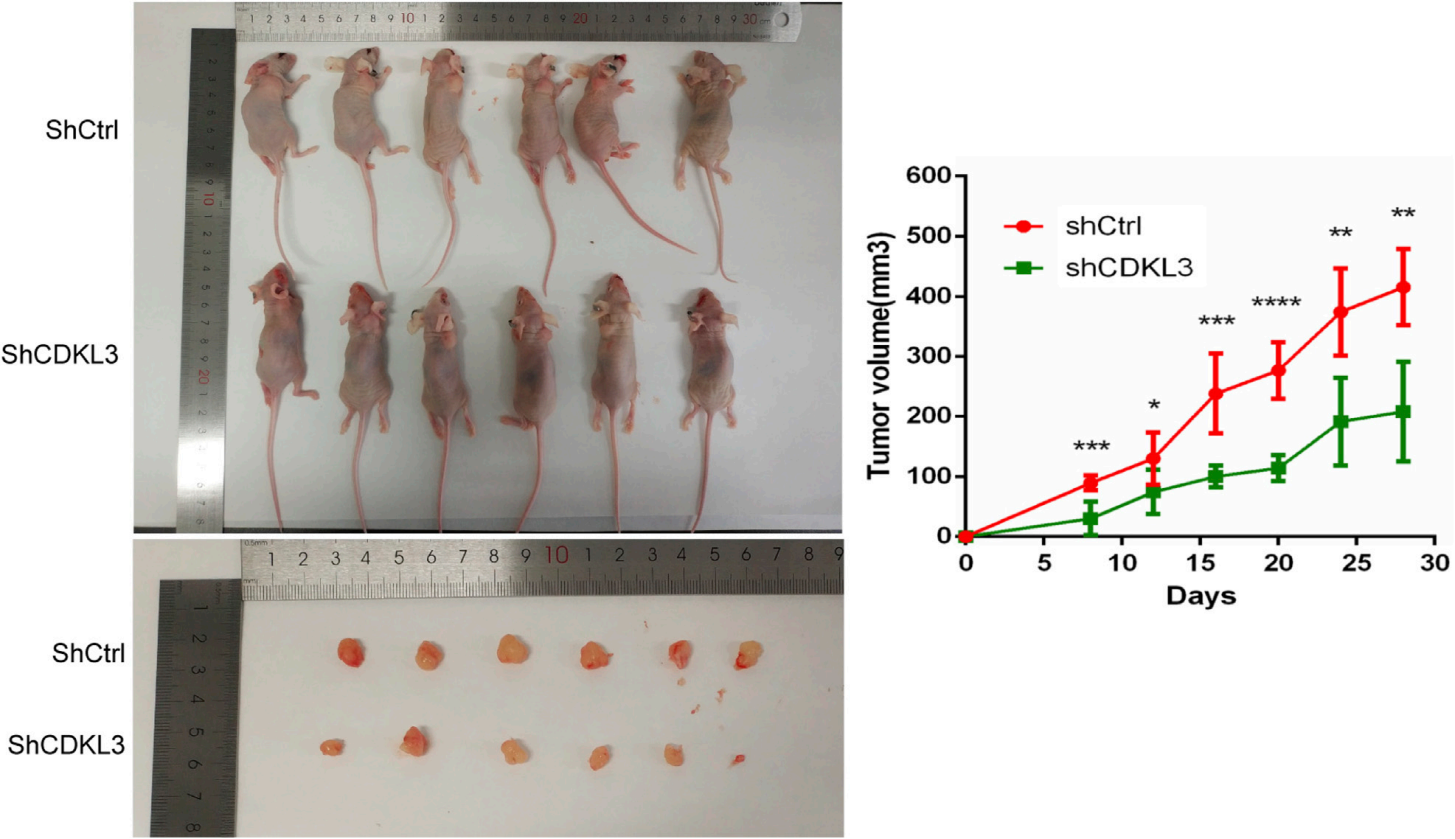
Knowdown of CDKL3 reduced tumor proliferation in xenografted nude mice. *p < 0.05, **p < 0.01, ***p < 0.005, ****p < 0.001. Error bars indicate mean ± SD.

To verify the effectiveness of CDKL3 knockdown, we performed qPCR and western blot assays, which confirmed the stable knockdown of CDKL3 in SMMC-7221 and HepG2 cells (Figures 7A, B). Through the CCK-8 assay, we were able to confirm that the knockdown of CDKL3 significantly reduced the proliferation of SMMC-7221 and HepG2 cells (Figure 7C). Furthermore, colony forming ability was also greatly inhibited in these cells as a result of CDKL3 knockdown (Figure 7D). Notably, the apoptosis rate in SMMC-7221 and HepG2 cells was markedly increased following CDKL3 knockdown, as demonstrated in Figure 8. Additionally, the migration of cells was significantly reduced upon CDKL3 knockdown (Supplementary Figure S12). Moreover, in the shCDKL3 group, the tumors formed by SMMC-7721 cells exhibited smaller volumes compared to those in the shCtrl group (Figure 9). These findings suggest that CDKL3 contributes to the tumorigenesis and advancement of HCC.

## Discussion

Recent studies have investigated the oncogenic role of CDKL3 in various tumors, including glioma [6], oesophageal squamous cell carcinoma [10], osteosarcoma [11], colorectal cancer [12], breast cancer [39], and cholangiocarcinoma [8]. However, the role of CDKL3 in HCC has not been thoroughly examined. In this study, we comprehensively analyzed the genetic characteristics and prognosis of CDKL3 using the LIHC dataset and confirmed its crucial function in the proliferation and progression of HCC.

According to pancancer analysis, the expression of CDKL3 is aberrant in different types of cancer, and it is higher in HCC tumor tissue compared to normal tissue. ROC analysis demonstrated that CDKL3 could serve as a potential diagnostic marker for distinguishing HCC from normal tissues. Furthermore, we found that high expression of CDKL3 is an independent predictor of poor prognosis. The nomogram that incorporates CDKL3 and independent clinicopathological factors demonstrated reliable performance in predicting prognosis. However, there have been limited studies that have focused on the genetic, TME, and metabolic features of CDKL3 in HCC biology using bioinformatic analysis. Our study fills this gap and highlights the significant genetic differences between the hCDKL3 group and the lCDKL3 group, including a higher occurrence of TP53 mutations, a greater burden of SCNA, a higher mRNAsi score, a higher PSI value of CDKL3−73367−AT, and a lower PSI value of CDKL3−73366−AT. Moreover, we elucidated the distinct TME and metabolic characteristics between the hCDKL3 and lCDKL3 groups. Additionally, we discovered that chemotherapeutic agents such as cisplatin, docetaxel, cytarabine, gemcitabine, bleomycin, paclitaxel, rapamycin, and sunitinib had higher IC50 values in the lCDKL3 group, suggesting that CDKL3 could potentially serve as a marker for diagnosing and predicting prognosis in HCC patients.

Consistent with previous findings showing that SCNA upregulation in tumors is linked to dismal patient survival [40], we observed that the hCDKL3 group had a higher level of SCNA and worse prognosis compared to the lCDKL3 group. Higher mRNAsi scores indicate active biological processes in cancer stem cells, which have been reported to contribute to tumor progression, recurrence, and therapeutic resistance [41]. Recently, evidence suggests that higher mRNAsi scores are related to a worse outcome for HCC patients [42]. Similarly, when comparing the hCDKL3 to the lCDKL3 group, we discovered that the former exhibited greater mRNAsi scores and a grim prognosis. Aberrant alternative splicing is closely linked to tumor proliferation, progression, prognosis, and therapeutic resistance [43, 44]. Changes in the expression of splicing factors can result in alterations in the alternative splicing of the target gene [43]. In our study, hCDKL3 with a higher PSI value of CDKL3−73367−AT and a lower PSI value of CDKL3−73366−AT may contribute to the posttranscriptional regulation of CDKL3, thereby leading to an unfavorable prognosis. Additionally, the hCDKL3 group exhibited increased expression of T-cell exhaustion markers and a worse prognosis. T-cell exhaustion has been shown to limit the anti-tumor response of the immune system and play a significant role in immune escape [45, 46]. Thus, the activation of T-cell exhaustion in hCDKL3 cells might contribute to a poorer prognosis. Overall, the distinct prognosis between the hCDKL3 and lCDKL3 groups is likely driven by the different genomic features of these two cohorts.

We undertook additional investigations to verify the functional relevance of CDKL3 in both *in vitro* and *in vivo* settings. Our results illustrated that suppressing CDKL3 expression contributed to a decline in cell proliferation, colony formation, and migration ability while inducing apoptosis in HCC cell lines. Additionally, *in vivo* experiments demonstrated a reduction in tumor volume upon the knockdown of CDKL3. These findings align with previous studies by Zhang et al., which highlighted curcumol-mediated inhibition of cholangiocarcinoma cell progression through CDKL3 knockdown [8], and Sun et al., whose research indicated that exosomal miRNA-205-5p from bone marrow mesenchymal stem cells could inhibit liver cancer, partially due to CDKL3 knockdown [19]. Our study strengthens and supports the antitumorigenic role of CDKL3.

Nevertheless, it is important to acknowledge several limitations in our present research. Firstly, despite using a public database to determine the prognostic implications of CDKL3, further investigations involving clinical samples are necessary to validate our results. Secondly, assessing the impact of CDKL3 on the immune microenvironment requires validation through molecular assays in future studies. Thirdly, additional biological experiments investigating the precise molecular mechanisms by which CDKL3 influences HCC progression, such as exploring the mechanistic pathways involved, are warranted.

## Conclusion

In conclusion, our findings suggest that CDKL3 may function as a significant molecular biomarker for diagnosing and estimating HCC prognosis. The observed tumor-inhibiting effect resulting from reduced CDKL3 expression indicates that CDKL3 may also be a promising molecular target for HCC therapy.

## Data Availability

The original contributions presented in the study are included in the article/Supplementary Material, further inquiries can be directed to the corresponding author.

## Footnotes

1
https://xena.ucsc.edu

2 Accessible at http://timer.comp-genomics.org/

3
https://www.gsea-msigdb.org

4
http://kobas.cbi.pku.edu.cn/

5
https://www.genepattern.org/

6
http://bioinformatics.mdanderson.org/TCGASpliceSeq/index.jsp
