# Supplementary material for: CDKL3 is a promising biomarker for diagnosis and prognosis prediction in patients with hepatocellular carcinoma
Source: Exp Biol Med (Maywood). 2024 Jun 27;249:10106. doi: 10.3389/ebm.2024.10106 (PMC11237920; doi:10.3389/ebm.2024.10106)
Supplement: Supplementary file 1 [file DataSheet1.pdf]

CDKL3 is a promising biomarker for diagnosis and prognosis prediction in patients with hepatocellular carcinoma

Running title: CDKL3 in HCC

Qingsi Wu[1,3*], Mengran Lu[4*], Huijuan Ouyang[4*], Tingting Zhou[4], Jingyuan Lei[4], Panpan Wang[4], Wei Wang[2]

[1]Department of Blood Transfusion, Second Affiliated Hospital of Anhui Medical University, Hefei, Anhui province, China, 230601; [2]Department of Gastroenterology, Yijishan Hospital of Wannan Medical College, Wuhu, Anhui province, China, 241000; [3]Anhui Provincial Key Laboratory of Microbiology & Parasitology, Hefei, Anhui province, China, 230032; [4]School of Public Health, Department of Hygiene Inspection and Quarantine, Anhui Medical University, Hefei, Anhui province, China, 230032.

[*]These authors contributed equally to this work and share first authorship

[*]**Correspondence:** Wei Wang (wwwy@wnmc.edu.cn)

ORCID: Wei Wang: 0000-0001-9963-5062

Supplementary Material and methods

Single-cell analysis

The Tumour Immune Single-Cell Hub (TISCH, http://tisch.comp-genomics.org) database was used to analyse the expression heterogeneity of CDKL3 in distinct immune cells based on the HCC single-cell dataset (GSE140228).[1]

Quantitative real-time polymerase chain reaction (qPCR) and western blot assays

For the qPCR assay, total RNA was extracted using TRIzol (Invitrogen, USA) and reverse-transcribed to cDNA using PrimeScript RT Reagent kits (Takara, Tokyo, Japan). Then, the relative CDKL3 mRNA expression was quantified using TB Green® Premix Ex Taq™ II Kit (Takara, Tokyo, Japan) according to the manufacturer's protocols. The relative expression level of CDKL3 was processed by the $2^{-\Delta\Delta Ct}$ method.[2] The primers used for CDKL3 were: forward: 5′-TCTTTCACCCCAGTGTGAGGT-3′; reverse: 5′-ACCTGGATCCTCTTGCCTGC-3′; for GAPDH were: forward: 5′-CGACCACTTTGTCAAGCTCA-3′; reverse: 5′-AGGGGTCTACATGGCAACTG-3′. For the western blot assay, total protein was extracted from HCC cell lines, followed by protein quantification using BCA Protein Assay Kit (Beyotime, Shanghai, China). 10% SDS-PAGE was used to separate protein samples. After transferring onto PVDF membranes (Millipore, Burlington, MA, United States), followed by blocking with 5% skim milk for 1 h and then separately incubated with mouse anti-CDKL3 antibody (Sigma-Aldrich, St. Louis, MO, United States) antibody (1:1000 diluted) overnight. Mouse anti-GAPDH (Santacruz, Santa Cruz, CA, United States) antibody (1:2000 diluted) was used as an internal control overnight (at 4°C). The visualization of protein bands was performed using a goat anti-mouse IgG secondary antibody (Santacruz, Santa Cruz, CA, United States).

Cell migration and wound healing assays

The infected cells that had been starved for 12 h were resuspended in serum-free medium and seeded into a transwell chamber (Corning Inc, Corning, NY) at a density of 20000 cells/well. The chamber was placed into a 24-well plate (Corning Incorporated, USA). After 12 h of incubation, we used a cotton swab to gently wipe the nonmigrated cells, and stained the cells with 0.1% crystal violet solution for 20 minutes (Beyotime, Shanghai, China).[3] The migrating cells were counted and photographed under a microscope. In the wound healing assay, the infected cells were seeded in a 12-well plate (Corning Incorporated, USA) to form a full monolayer. The wounds were created by scratching using 200 μL pipette tips, and the intercellular distance was detected at 0 h, 12 h and 24 h.

Competitive endogenous RNA (ceRNA) regulatory networks

Differential expression analyses between HCC and controls were performed to obtain the differentially expressed genes (DEGs) using the "limma" package, which determined the thresholds for DEmiRNAs (logFC < -0.5 and FDR < 0.01) and DElncRNAs (FDR < 0.01). We chose DEmiRNAs that had a significant negative correlation with CDKL3 ($r < -0.1$ and $P < 0.001$) and complied with the prediction results from TargetScan (http://www.targetscan.org/vert_72/), as the potential miRNAs interacted with CDKL3.[4] LncBase Predicted v2 (http://carolina.imis.athena-innovation.gr/diana_tools) was used to predict lncRNA–miRNA pairs (score > 0.9).[5] The potential lncRNAs interacting with miRNAs met the following criteria: 1. intersection with DElncRNAs; 2. significant negative correlation with the above potential miRNAs. Finally, we constructed and visualized ceRNA networks using Cytoscape (version 3.7.0, https://www.cytoscape.org/).

Estimation of the benefit from immunotherapy and chemotherapy

The estimation of immunotherapy response was performed using the Tumour Immune Dysfunction and Exclusion (TIDE) algorithm.[6] The chemotherapy response was evaluated using the "pRRophetic" package to calculate the half-maximal inhibitory concentration (IC50) values based on the data of Genomics of Drug Sensitivity in Cancer (GDSC, https://www.cancerrxgene.org/) according to previously described methods.[7] HCC patients who possessed higher estimated IC50 values had lower rates of therapy benefit from the given compound.[8]

Supplementary Results

Mutation features between the hCDKL3 and lCDKL3

As shown in Supplementary Fig. 4A, in hCDKL3, almost half of the patients (42%) had TP53 mutations, which was significantly higher than the rate for the lCDKL3 group. However, we found no significant differences in the mutation counts (nonsynonymous, synonymous and all) between the hCDKL3 and lCDKL3 groups (Supplementary Fig. 5A). In addition, no significant differences were detected between the hCDKL3 and lCDKL3 groups in terms of TMB and methylation level of the promoter (Supplementary Fig. 5B-D). We next compared the SCNA level and observed that the hCDKL3 group had a higher rate of copy number gain or loss than the lCDKL3 group at both the focal and arm levels (Supplementary Fig. 4B).

Tumour immune single-cell analysis

In terms of single-cell analysis in the TISCH database, GSE140228 was classified into 10 types of cells and 20 cell clusters (Supplementary Fig. 9A); the most number enriched cell type was monocyte/macrophage cells, followed by NK cells, $CD4^{+}FoxP3^{-}$ T cells (CD4Tconv), CD8+T exhaustion cells (CD8Tex) and B cells (Supplementary Fig. 9B). As shown in Supplementary Fig. 3C and D, CDKL3 was correlated with a high infiltration level in CD4Tconv, NK cells and B cells, followed by CD8Tex, monocyte/macrophage cells and innate lymphoid cells (ILCs).

Construction of the ceRNA regulatory network based on CDKL3

We first identified three potential miRNAs interacting with CDKL3 in the TCGA dataset according to the criteria described in the Methods section. Then, we used the TargetScan algorithm to screen only one matched miRNA (hsa-miR-139-3p). A total of 18 lncRNAs were predicted to interact with hsa-miR-139-3p, in which 4 lncRNAs (AC006273.4, KCNQ1OT1, PCAT6 and RP11-386G11.5) met the criteria described in the Methods section as potential LncRNAs to construct the ceRNA networks (Supplementary Fig. 10).

Identification of potential therapeutic agents

As shown in Supplementary Fig. 11A, more immunotherapy responders were found in hCDKL3 than in lCDKL3 using ImmuneCellAI algorithm analysis, but the difference did not reach statistical significance. As Supplementary Fig. 11B, the majority of chemotherapeutics had higher IC50 values in the lCDKL3 group; these included cisplatin, docetaxel, cytarabine, gemcitabine, bleomycin, paclitaxel, rapamycin and sunitinib. However, the IC50 values of axitinib were higher in the hCDKL3 group.

References

1. Sun D, Wang J, Han Y, Dong X, Ge J, Zheng R, Shi X, Wang B, Li Z, Ren P, Sun L, Yan Y, Zhang P, Zhang F, Li T, Wang C. TISCH: a comprehensive web resource enabling interactive single-cell transcriptome visualization of tumor microenvironment. *Nucleic Acids Res* 2021;**49**:D1420-30.
2. Jin S, Li X, Dai Y, Li C, Wang D. NF-kappaB-mediated miR-650 plays oncogenic roles and activates AKT/ERK/NF-kappaB pathways by targeting RERG in glioma cells. *Cell Oncol* 2020;**43**:1035-48.
3. Wei S, Peng L, Yang J, Sang H, Jin D, Li X, Chen M, Zhang W, Dang Y, Zhang G. Exosomal transfer of miR-15b-3p enhances tumorigenesis and malignant transformation through the DYNLT1/Caspase-3/Caspase-9 signaling pathway in gastric cancer. *J Exp Clin Cancer Res* 2020;**39**:32.
4. Agarwal V, Bell GW, Nam JW, Bartel DP. Predicting effective microRNA target sites in mammalian mRNAs. *Elife* 2015;**4**:e05005
5. Paraskevopoulou MD, Vlachos IS, Karagkouni D, Georgakilas G, Kanellos I, Vergoulis T, Zagganas K, Tsanakas P, Floros E, Dalamagas T, Hatzigeorgiou AG. DIANA-LncBase v2: indexing microRNA targets on non-coding transcripts. *Nucleic Acids Res* 2016;**44**(D1):D231-8
6. Jiang P, Gu S, Pan D, Fu J, Sahu A, Hu X, Li Z, Traugh N, Bu X, Li B, Liu J, Freeman GJ, Brown MA, Wucherpfennig KW, Liu XS. Signatures of T cell dysfunction and exclusion predict cancer immunotherapy response. *Nat Med* 2018;**24**:1550–8
7. Yang W, Soares J, Greninger P, Edelman EJ, Lightfoot H, Forbes S, Bindal N, Beare D, Smith JA, Thompson IR, Ramaswamy S, Futreal PA, Haber DA, Stratton MR, Benes C, McDermott U, Garnett MJ. Genomics of Drug Sensitivity in Cancer (GDSC): a resource for therapeutic biomarker discovery in cancer cells. *Nucleic Acids Res* 2013;**41**:D955–61
8. Geeleher P, Cox N, Huang RS. pRRophetic: an R package for prediction of clinical chemotherapeutic response from tumor gene expression levels. *PloS One* 2014;**9**:e107468

Supplemental Table 1 Clinicopathological Characteristics of the HCC patients in TCGA

**Characteristics**	**Patients 330 (100%)**	**Characteristics**	**Patients 330 (100%)**
**Age**		**Family cancer history**	
<= 60	160(48.5%)	no	187 (56.7%)
>60	170(51.5%)	yes	101 (30.6%)
**Sex**		Unknown	42 (12.7%)
Female	105 (31.8%)	**Cancer status**	
Male	225 (68.2%)	tumor free	206 (62.4%)
**Race**		with tumor	103 (31.2%)
White	157 (47.6%)	Unknown	21 (6.4%)
Asian	147 (44.5%)	**Stage**	
other	16 (4.9%)	1+2	227 (68.8%)
Unknown	10 (3.0%)	3+4	81 (24.5%)
**BMI**		Unknown	22 (6.7%)
<18.5	18 (5.5%)	**Child pugh**	
18.5-25	140 (42.4%)	A	198 (60.0%)

>=25	148 (44.8%)	B+C	20 (6.1%)
Unknown	24 (7.3%)	Unknown	112 (33.9%)
**Grade**		**Fibrosis ishak score**	
1+2	205 (62.1%)	0	67 (20.3%)
3+4	120 (36.4%)	1-4	54 (16.4%)
Unknown	5 (1.5%)	5-6	70 (21.2%)
**residual tumor**		Unknown	139 (42.1%)
R0	294 (89.1%)		
R1+R2	14 (4.2%)		
Unknown	22 (6.7%)		

Supplemental Table 2 The most strongly 50 positive and 50 negative correlated co-expressed genes with CDKL3 based on the TCGA data

Gene	cor	pvalue
RBM22	0.521	0
ZNF346	0.52	0
BRD8	0.497	0
ZCCHC10	0.471	0
CWC27	0.464	0
GRPEL2	0.462	0
MKS1	0.462	0
TIPIN	0.462	0
BOD1	0.459	0
IK	0.457	0
PPP2CA	0.457	0
CDKN2AIPNL	0.454	0
SKP1	0.453	0
POLG2	0.449	0
C12orf43	0.446	0
FASTKD3	0.446	0
HARS2	0.445	0
LARS	0.444	0
C15orf40	0.442	0

RECQL5	0.442	0
CSTF3	0.44	0
MED7	0.439	0
ZFP62	0.439	0
C2orf44	0.438	0
CETN3	0.436	0
IFT172	0.436	0
TARDBP	0.436	0
DROSHA	0.435	0
UIMC1	0.435	0
MRPS27	0.434	0
SLU7	0.434	0
TTC23L	0.433	1.63E-16
WDR41	0.433	0
ERCC8	0.432	0
GPBP1	0.43	0
C17orf80	0.429	0
SF3A3	0.429	0
WDR70	0.429	0
DZANK1	0.425	0
GIN1	0.425	0
JRK	0.425	0
WDR4	0.425	0
AMZ2	0.423	0
DNAJC7	0.423	0
ZNHIT3	0.423	0
CCDC30	0.422	0
FAF2	0.422	0
CDC23	0.421	0
C5orf45	0.42	0
KCTD2	0.42	0
APOC3	-0.283	1.90E-07
SERPINA11	-0.27	7.40E-07

F9	-0.268	7.96E-07
CA5A	-0.252	3.71E-06
HPX	-0.25	4.45E-06
CFHR3	-0.243	8.22E-06
NLRP6	-0.243	7.69E-06
CFHR4	-0.235	1.67E-05
ELFN1	-0.228	3.00E-05
CFHR1	-0.227	3.23E-05
MT-ND6	-0.222	4.95E-05
MASP2	-0.219	6.40E-05
APOA5	-0.218	7.01E-05
RBP4	-0.217	7.36E-05
CPB2	-0.216	8.07E-05
HP	-0.216	8.18E-05
C8B	-0.213	0.000101208
SERPING1	-0.213	9.62E-05
ECHS1	-0.209	0.000134257
LEAP2	-0.207	0.000155237
LIMS2	-0.207	0.000162288
HPR	-0.205	0.000177614
ADH4	-0.204	0.000190275
C4BPA	-0.204	0.000195827
FBP1	-0.204	0.000202544
SERPINA10	-0.204	0.000198026
SULT2A1	-0.204	0.0001908
TGFBR3L	-0.204	0.000189726
AZGP1	-0.202	0.000233121
PROC	-0.202	0.000227917
PEMT	-0.201	0.000244176
SLC10A1	-0.2	0.00027044
SLC27A5	-0.199	0.000279736
ANG	-0.198	0.000294111
RGN	-0.198	0.000292938

APCS	-0.197	0.000321504
CLEC3B	-0.197	0.000320618
G0S2	-0.197	0.000320016
RAMP3	-0.197	0.000317348
APOC1	-0.196	0.00036109
BAAT	-0.196	0.000349787
ALDOB	-0.192	0.000473277
AFM	-0.191	0.000494214
MT-CO1	-0.19	0.000516876
PROL1	-0.189	0.000568169
HSD11B1	-0.188	0.000591458
RIPPLY1	-0.188	0.000611289
TM6SF2	-0.188	0.000610368
UROC1	-0.186	0.000712953
HFE2	-0.185	0.000730763

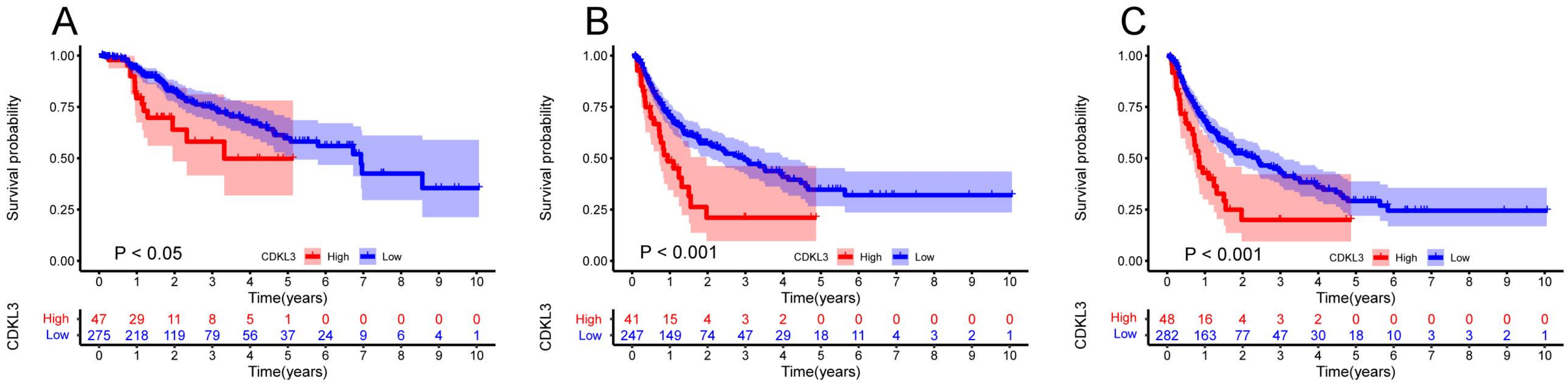

Fig. S1 Kaplan-Meier survival curves of hCDKL3 and lCDKL3 and the survival difference was evaluated by log-rank test. (A) disease-specific survival. (B) disease-free interval. (C) progression-free interval

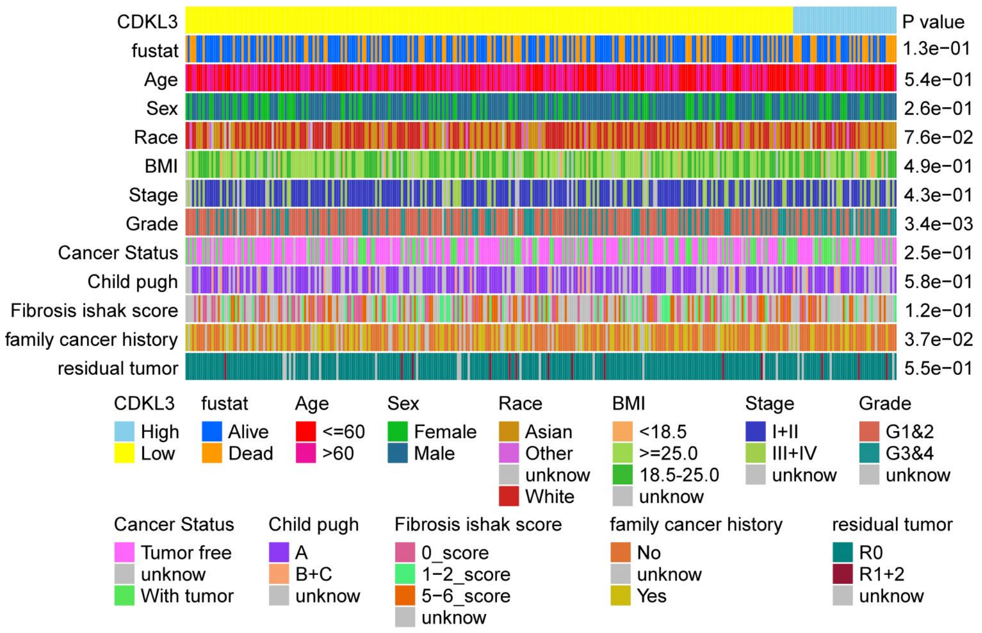

Fig. S2 Clinicopathological characteristics in hCDKL3 and lCDKL3.

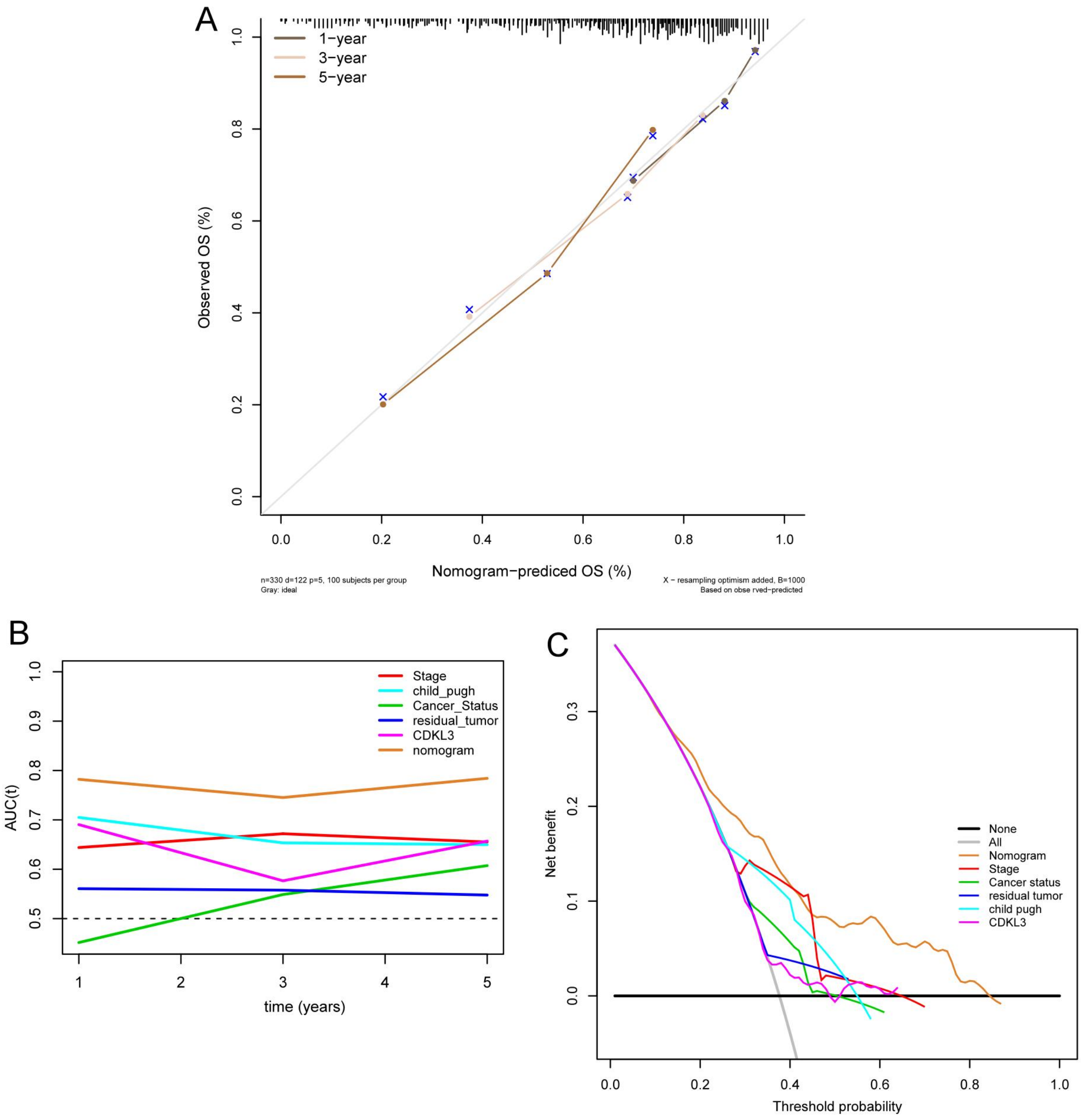

Fig. S3 (A) Calibration plots, (B) Time-dependent ROC analysis and the AUC at 1, 3, and 5-years, and (C) Decision curve analysis of nomogram for predicting overall survival of HCC in TCGA cohort.

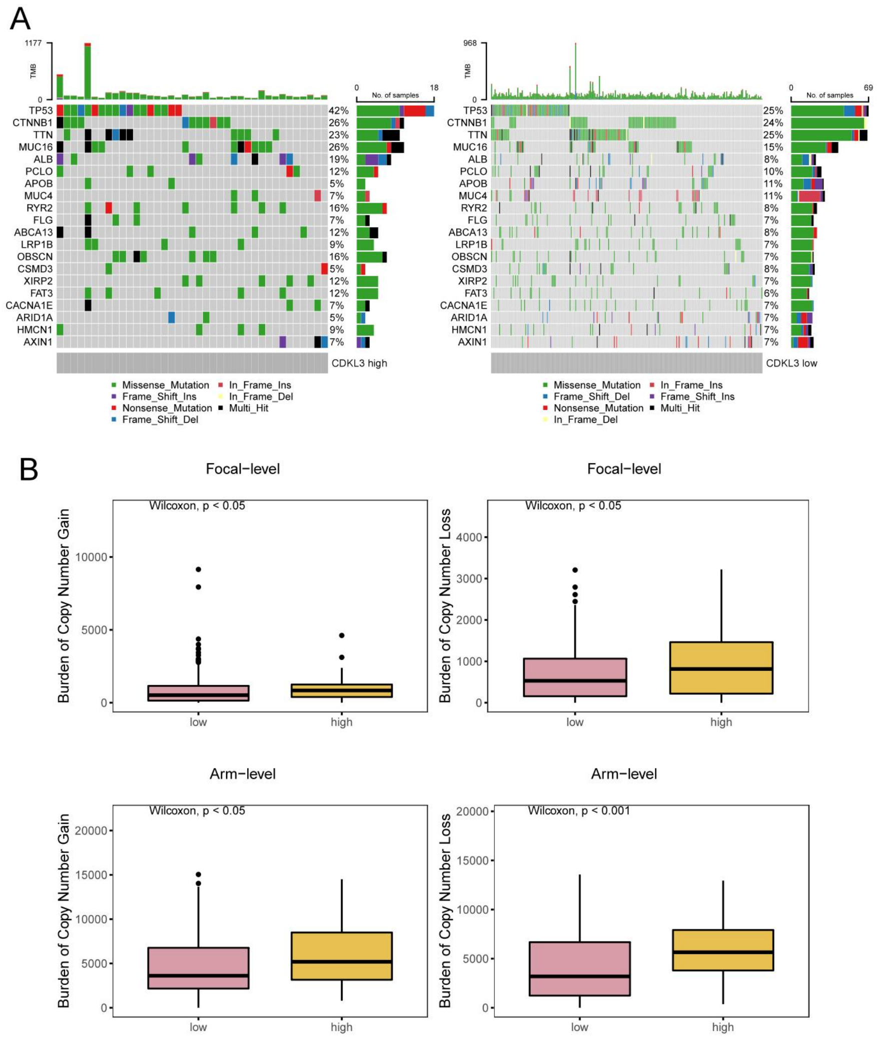

Fig. S4 (A) Somatic mutation landscapes and (B) burden of somatic copy number alternation in hCDKL3 and lCDKL3.

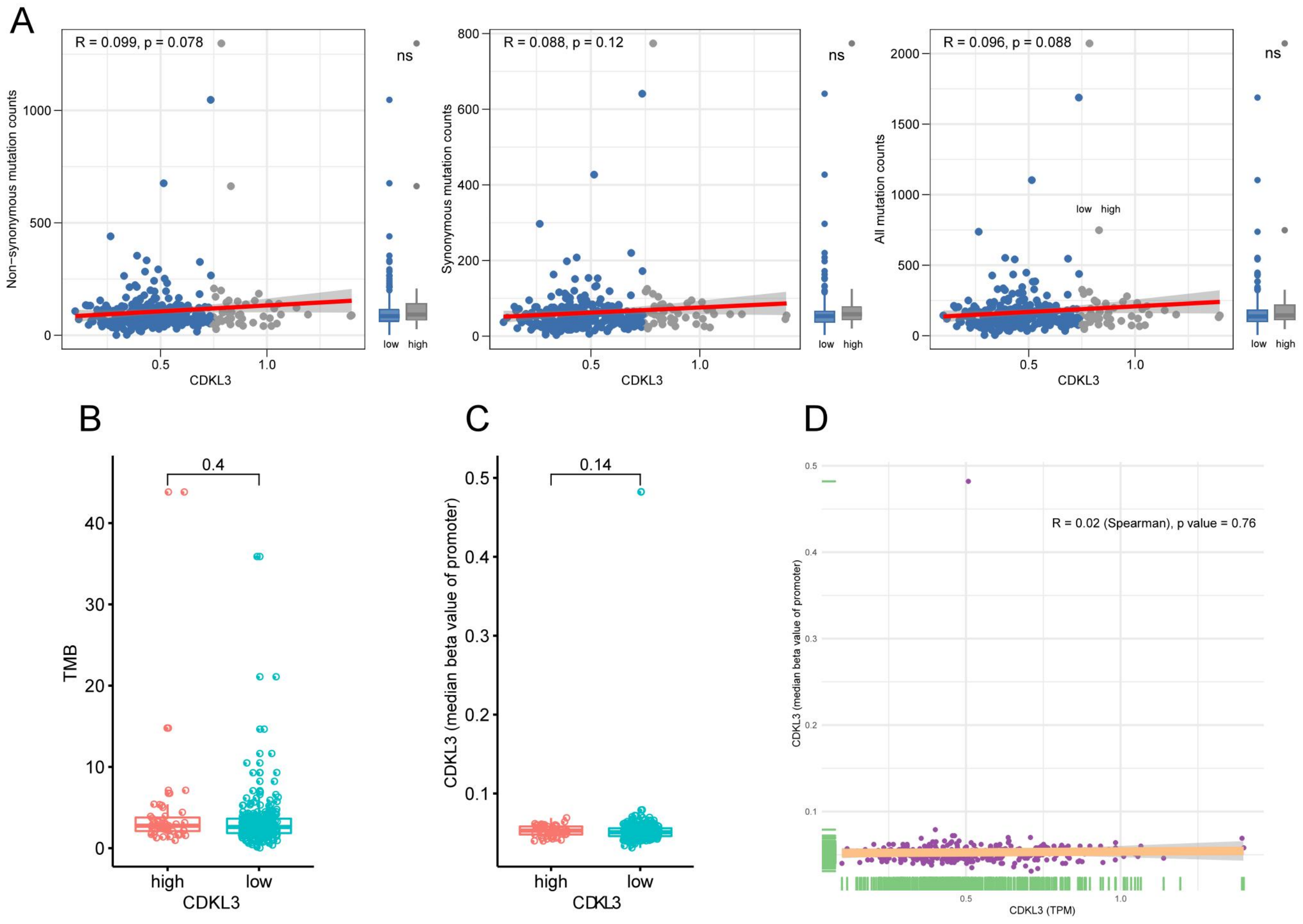

Fig. S5 The features of mutation counts, TMB and methylation level of promoter were showed in HCC. (A) The relationship between CDKL3 expression and mutation counts. (B) The TMB was compared between hCDKL3 and lCDKL3. (C) The methylation level of promoter was compared between hCDKL3 and lCDKL3. (D) The relationship between CDKL3 expression and methylation level of promoter.

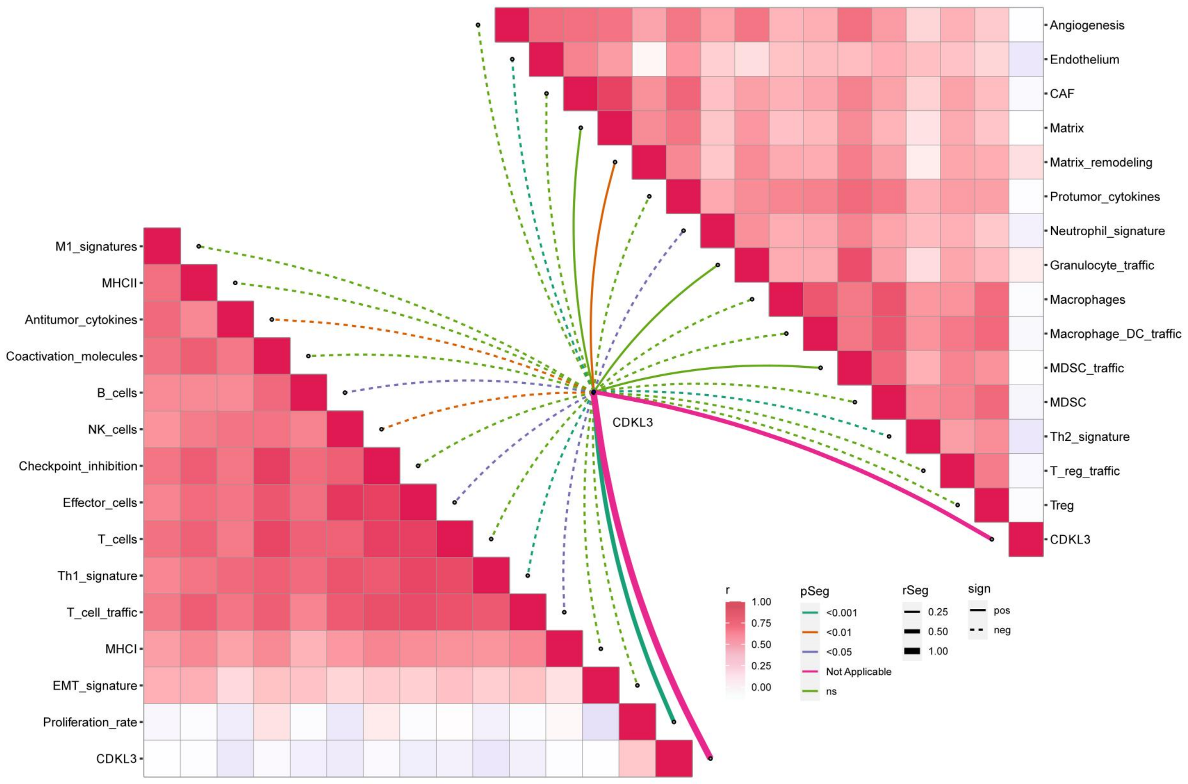

Fig. S6 The correlation analysis of the CDKL3 expression and enrichment score of 29 Fges.

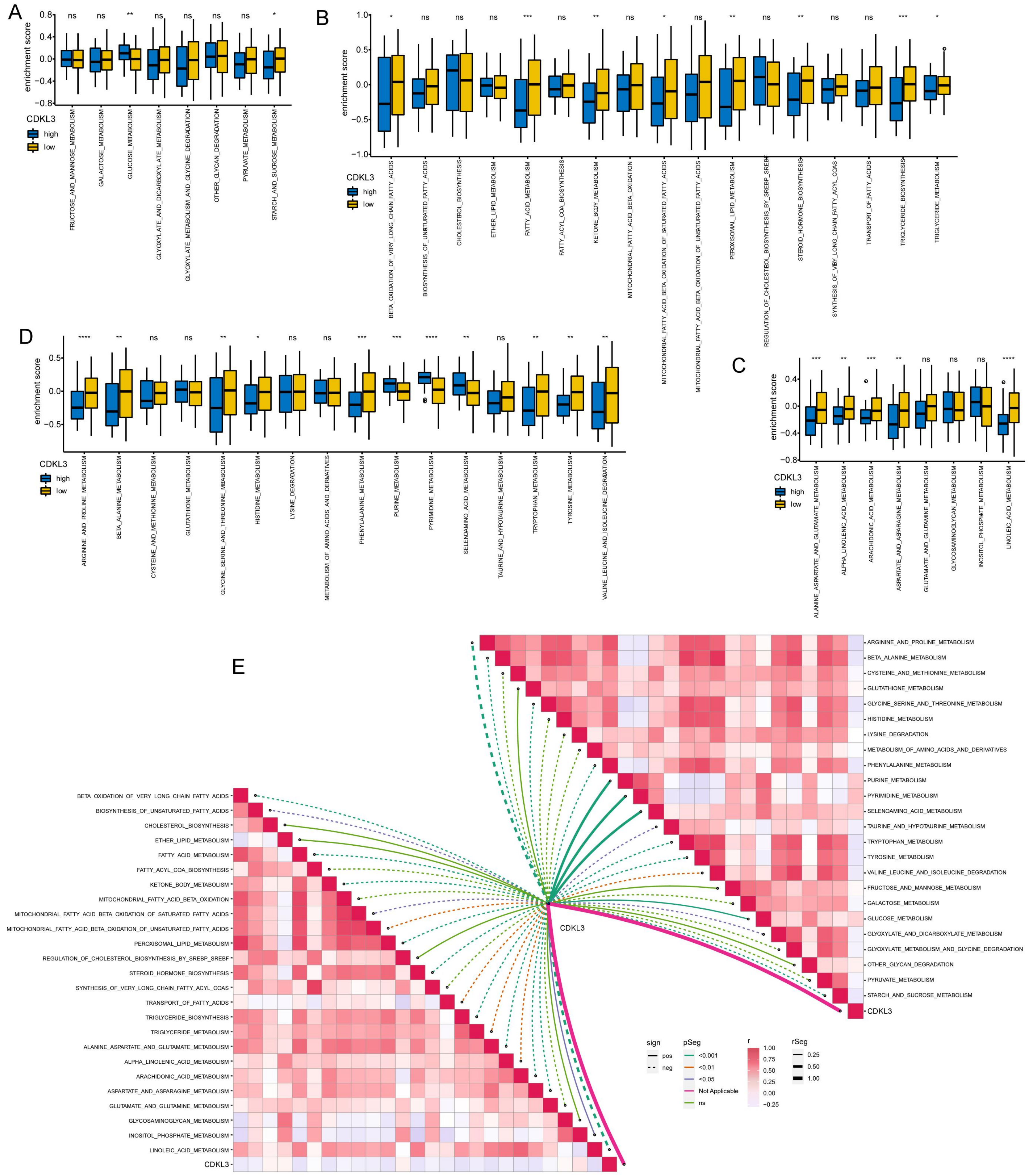

Fig. S7 The metabolic characteristics of hCDKL3 and lCDKL3. The enrichment scores of metabolic-related pathways in hCDKL3 and lCDKL3. (A) Carbohydrate metabolism. (B) Lipid metabolism. (C) Others metabolism. (D) Amino acid metabolism. (E) The correlation analysis of the CDKL3 expression and enrichment scores of metabolic-related pathways. *P < 0.05, **P < 0.01, ***P < 0.001, ****P < 0.0001, ns: not significantly significant.

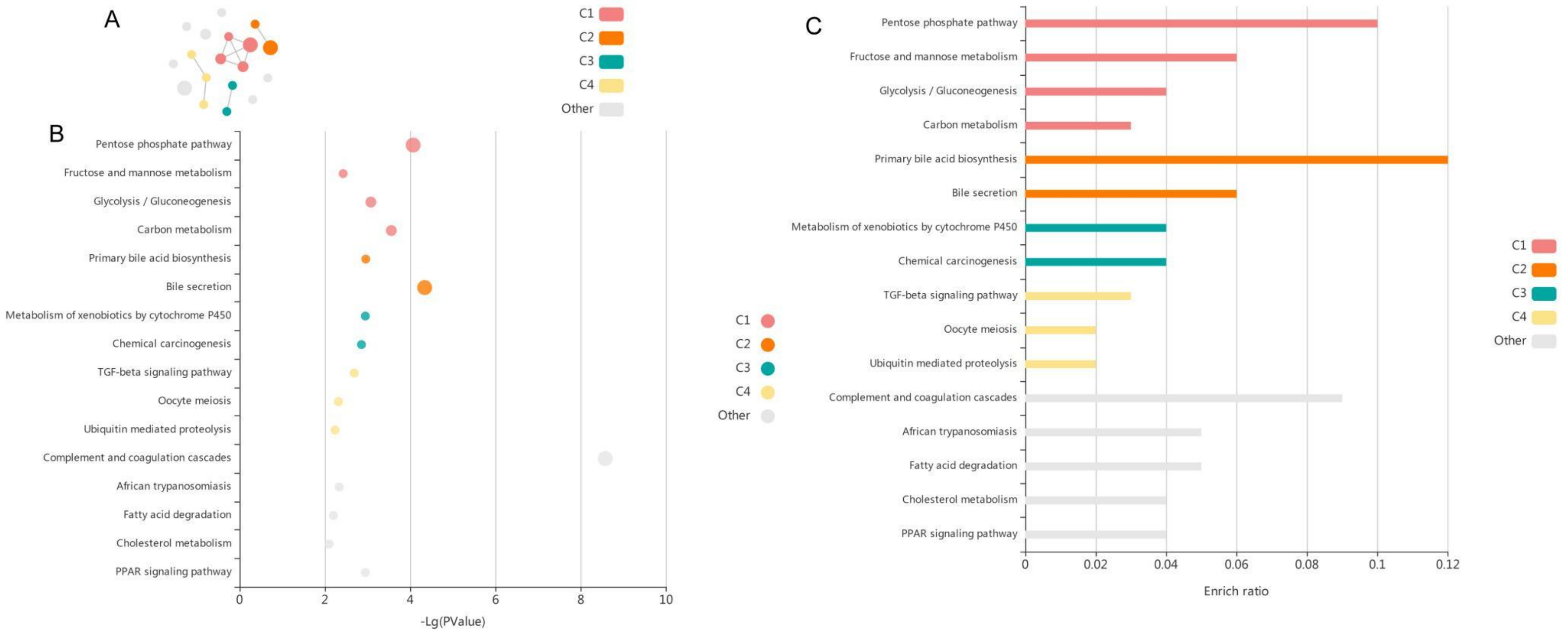

Fig. S8 Functional enrichment KEGG pathways using KOBAS-i (http://kobas.cbi.pku.edu.cn/) based on the most strongly 100 correlated co-expressed genes with CDKL3. (A) Enriched terms visualized in cirFunMap. Each node represents an enriched term, and the node color represents different clusters; the node size represents 6 levels of enriched p-value, node size from small to large: [0.05, 1], [0.01, 0.05), [0.001, 0.01], [0.0001, 0.001], [1e-10, 0.0001], [0, 1e-10]; the edge represents correlations larger than the user-defined threshold. (B) Enriched terms visualized in bubble plot. Each bubble represents an enriched function, and the size of the bubble from small to large: [0.05, 1], [0.01, 0.05], [0.001, 0.01], [0.0001, 0.001], [1e-10, 0.0001], [0, 1e-10]. The color of the bar is the same as the color in the circular network, which represents different clusters. For each cluster, if there are more than 5 terms, top 5 with the highest enrich ratio will be displayed. (C) Enriched terms visualized in barplot. Each row represents an enriched function, and the length of the bar represents the enrich ratio, which is calculated as “input gene number”/ “backgound gene number”. The color of the bar is the same as the color in the circular network in above, which represents different clusters. For each cluster, if there are more than 5 terms, top 5 with the highest enrich ratio will be displayed.

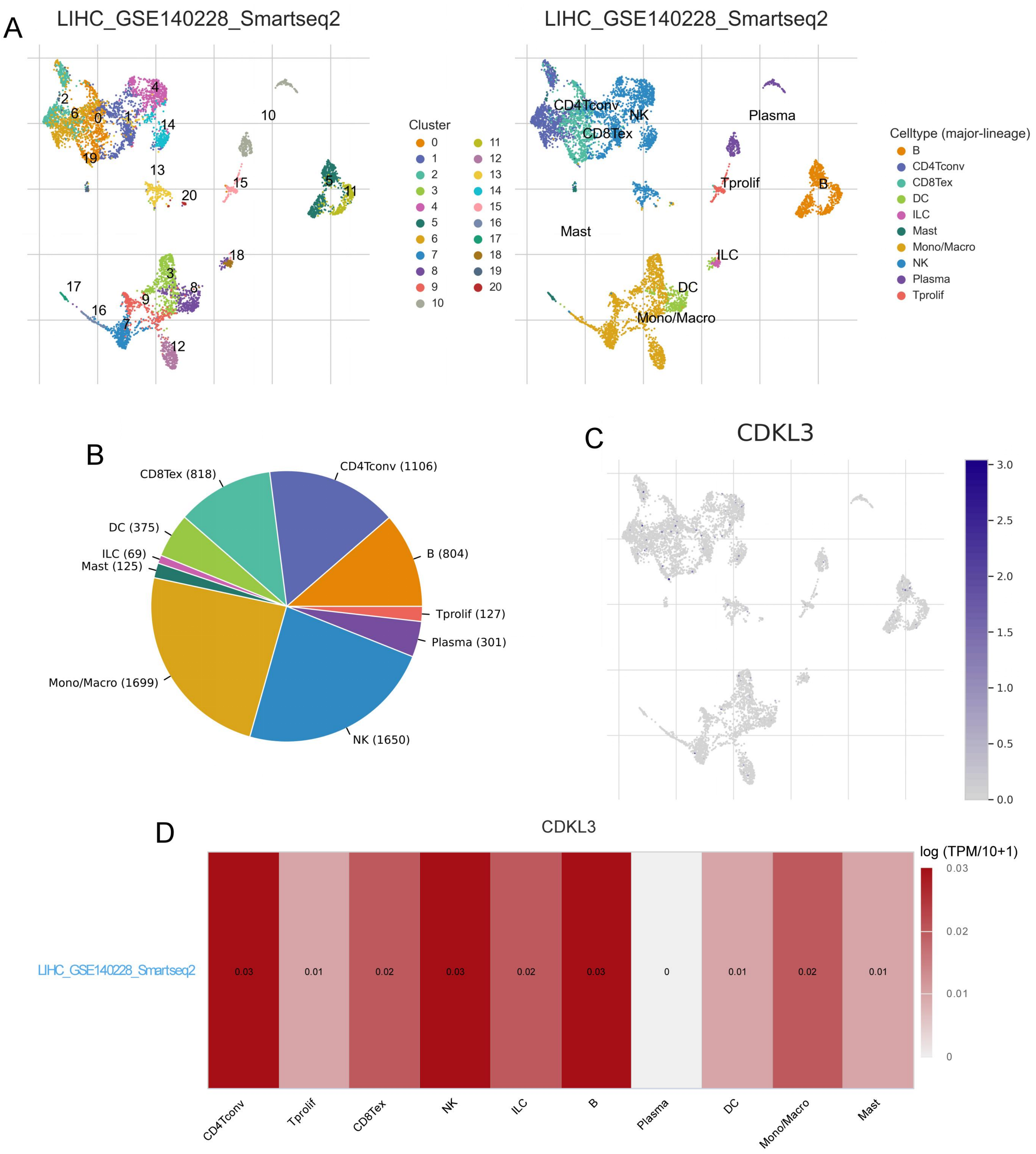

Fig. S9 Expression of CDKL3 in tumor microenvironment-related cells (TISCH). (A) Annotation of all cell types in GSE140228 and (B) the percentage of each type of cell. (C) The proportion of CDKL3 in GSE140228 in various types of cells. (D) The expression level of CDKL3 in the tumor microenvironment-related cells of HCC in GSE140228.

1

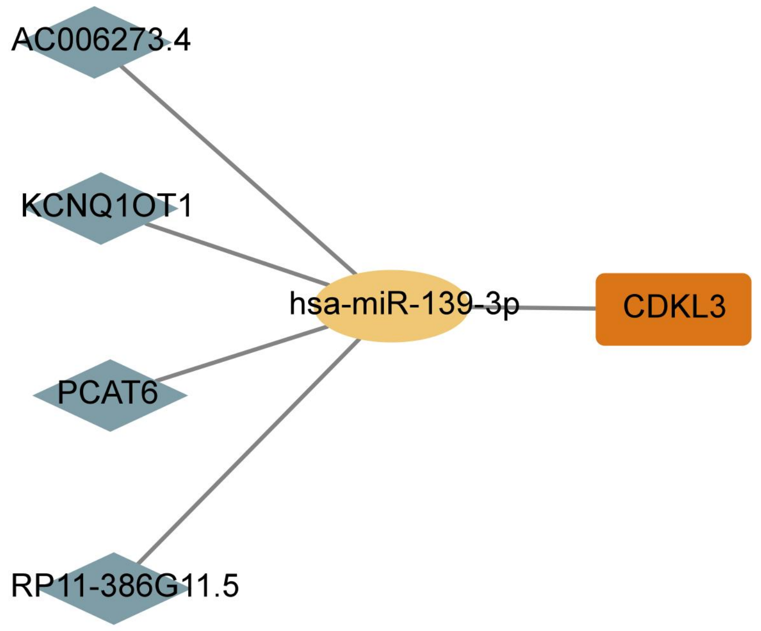

Fig. S10 Construction of the ceRNA regulatory network. The ellipses denote miRNA, diamonds denote lncRNAs, and round rectangle denote mRNA.

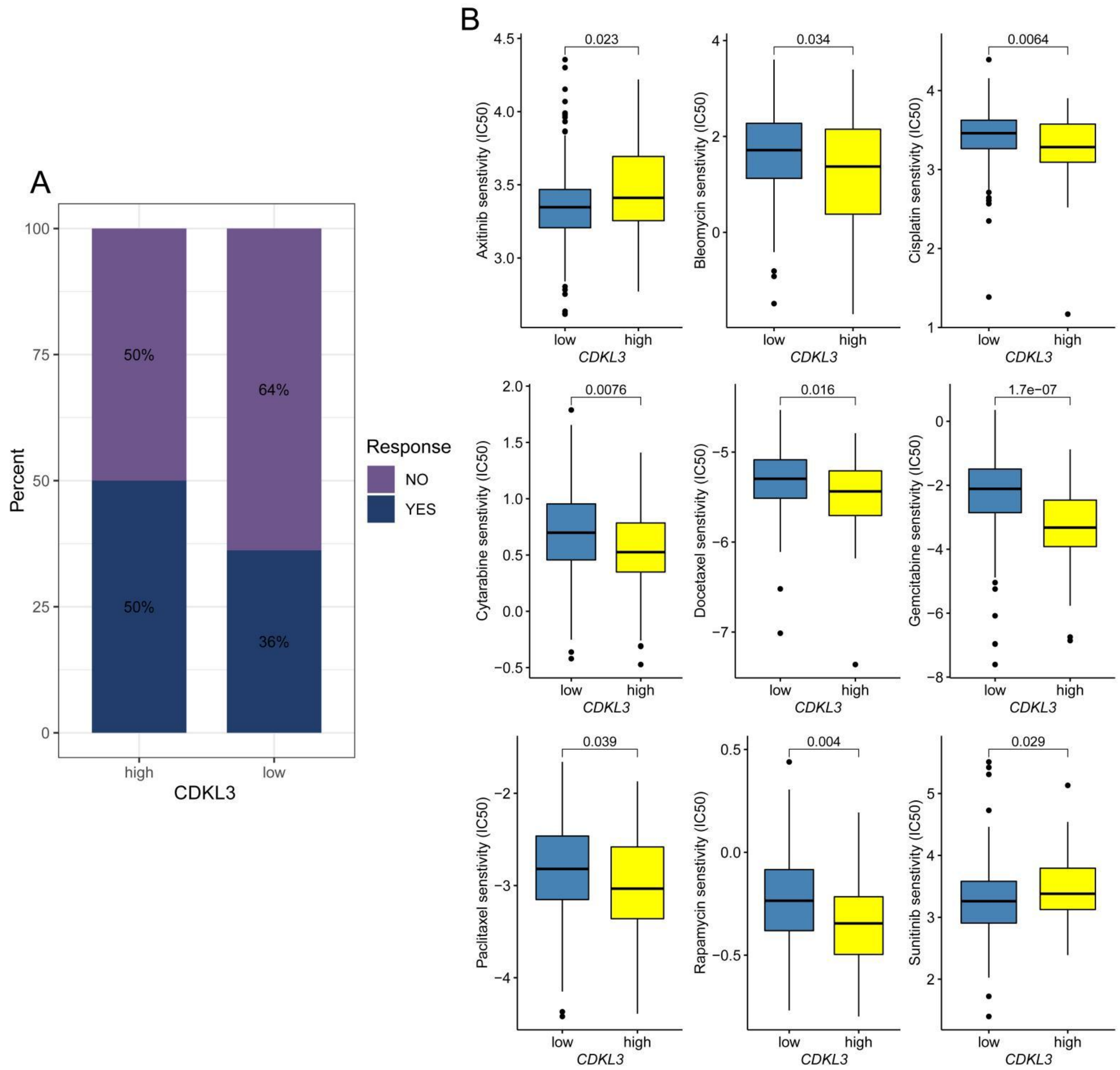

Fig. S11 Differential immunotherapeutic and chemotherapeutic response. (A) The proportion of patients with response to immunotherapy in hCDKL3 and lCDKL3 calculated by ImmuneCellAI. (B) Box plots for the estimated IC50 of chemotherapy drugs in hCDKL3 and lCDKL3.

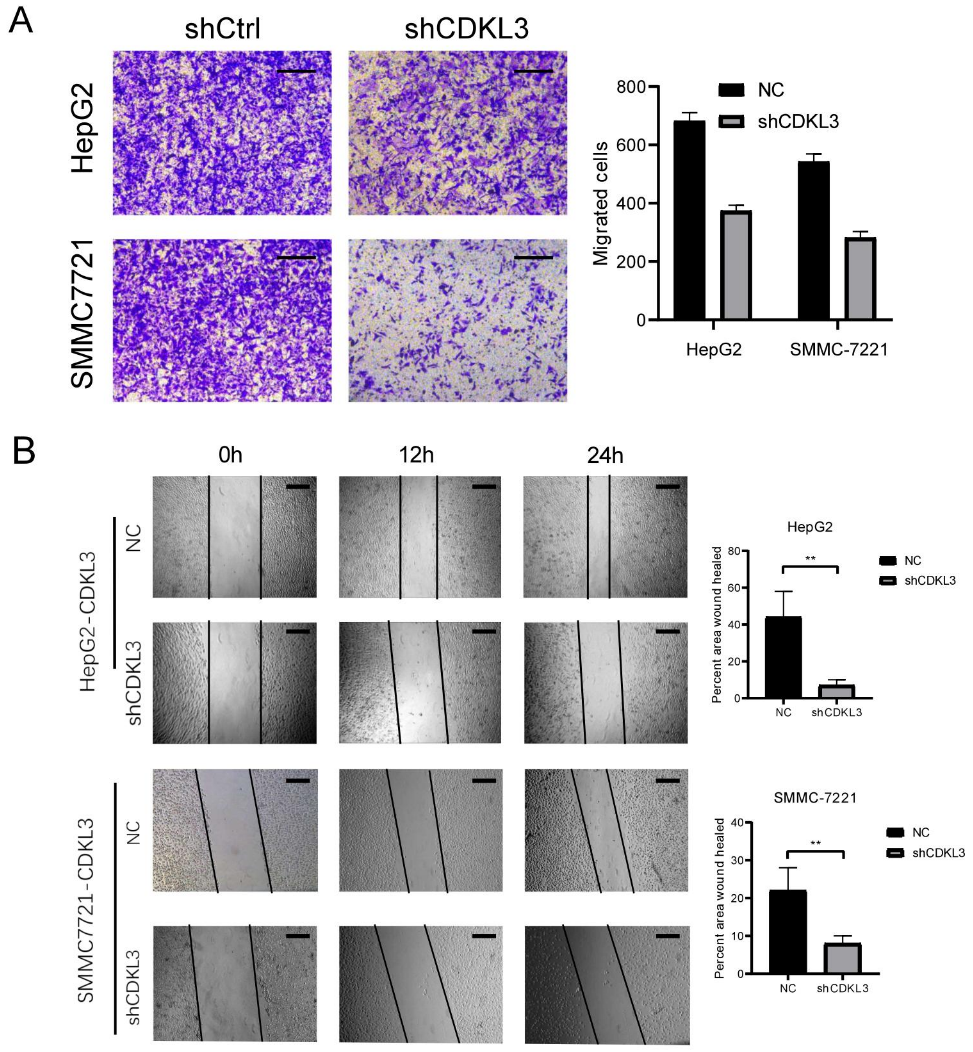

Fig. S12 Transwell (Scale bar, 300μm) and wound healing (Scale bar, 200μm) assays were performed to evaluate cell migration ability. **P < 0.01. Error bars indicate mean± SD.
